# Plant evolution in alkaline magnesium-rich soils: A phylogenetic study of the Mediterranean genus *Hormathophylla* (Cruciferae: Alysseae) based on nuclear and plastid sequences

**DOI:** 10.1371/journal.pone.0208307

**Published:** 2018-12-21

**Authors:** Esteban Salmerón-Sánchez, Javier Fuertes-Aguilar, Stanislav Španiel, Francisco Javier Pérez-García, Encarna Merlo, Juan Antonio Garrido-Becerra, Juan Mota

**Affiliations:** 1 Departamento de Biología y Geología, CEI.MAR and CECOUAL, Universidad de Almería, Almería, Spain; 2 Departamento de Botánica, Unidad de Conservación Vegetal, Universidad de Granada, Granada, Spain; 3 Real Jardín Botánico, CSIC, Madrid, Spain; 4 Institute of Botany, Plant Science and Biodiversity Centre, Slovak Academy of Sciences, Bratislava, Slovak Republic; 5 Department of Botany, Faculty of Science, Charles University in Prague, Prague, Czech Republic; Seoul National University College of Medicine, REPUBLIC OF KOREA

## Abstract

Habitats with alkaline edaphic substrates are often associated with plant speciation and diversification. The tribe Alysseae, in the family Brassicaceae, epitomizes this evolutionary trend. In this lineage, some genera, like *Hormathophylla*, can serve as a good case for testing the evolutionary framework. This genus is centered in the western Mediterranean. It grows on different substrates, but mostly on alkaline soils. It has been suggested that diversification in many lineages of the tribe Alysseae and in the genus *Hormathophylla* is linked to a tolerance for high levels of Mg^+2^ in xeric environments. In this study, we investigated the controversial phylogenetic placement of *Hormathophylla* in the tribe, the generic limits and the evolutionary relationships between the species using ribosomal and plastid DNA sequences. We also examined the putative association between the evolution of different ploidy levels, trichome morphology and the type of substrates. Our analyses demonstrated the monophyly of the genus *Hormathophylla* including all previously described species. Nuclear sequences revealed two lineages that differ in basic chromosome numbers (x = 7 and x = 8 or derived 11, 15) and in their trichome morphology. Contrasting results with plastid genes indicates more complex relationships between these two lineages involving recent hybridization processes. We also found an association between chloroplast haplotypes and substrate, especially in populations growing on dolomites. Finally, our dated phylogeny demonstrates that the origin of the genus took place in the mid-Miocene, during the establishment of temporal land bridges between the Tethys and Paratethys seas, with a later diversification during the upper Pliocene.

## Introduction

The family Brassicaceae Burnett is composed of about 49 tribes, 321 genera and 3,600 species with a worldwide distribution [1], mainly in regions with temperate climates [2–3]. The tribal classification [4–7] based mainly on morphological characters (orientation of the radicle in the embryo, relative size and degree of compression of the fruit, dehiscence, number of seed rows per loculus, trichomes and nectaries types), have revealed a high level of homoplasy thanks to contributions from molecular phylogenetics. Therefore, the family has undergone major reorganization including the intrafamilial categories [3,8–15].

The recently revised tribe Alysseae DC. [16–17], although reasonably well delimited, still presents some conflicting evidence from a phylogenetic perspective [1–3,16], that only recently has been partially solved [17]. According to Španiel et al. [17], the tribe currently includes 24 genera, with a distribution mainly centered in Eurasia and North Africa, encompassing 277 species of which more than a half belong to the genus *Alyssum* L. (114 spp.). The following genera by number of species are *Odontarrhena* C.A.Mey. ex Ledeb. (87 spp.) and *Hormathophylla* Cullen & T.R.Dudley (10 spp.) [18]. All members of Alysseae are characterized by stellate trichomes, latiseptate or cylindrical (rarely angustiseptate) fruits, with few seeds, winged seeds and a basic chromosome number of x = 8, although smaller or larger derived numbers have also been observed [17] (*Alyssum*, *Odontarrhena*, *Clypeola*, *Hormathophylla*). *Hormathophylla* has been the subject of several taxonomic treatments since its original description [19–23] and, although current evidence suggests that *Hormathophylla* is a monophyletic genus [3,16], these studies are based on a limited sampling of species omitting several taxa of controversial identity.

**Fig 1:**
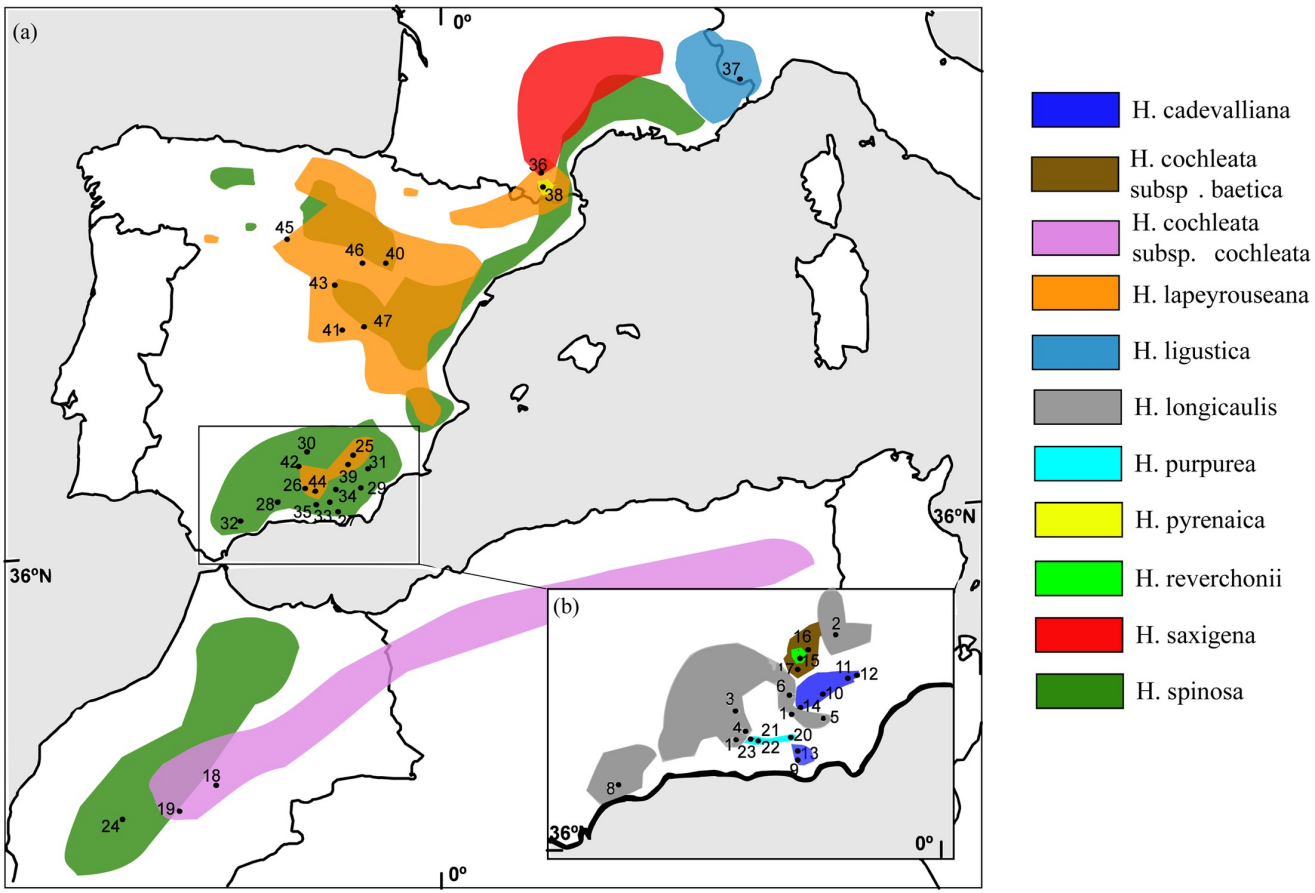
Distribution area of 11 taxa in *Hormathophylla*. Sampled localities are represented by dots and numbers as detailed in Table 1. A) *Hormathophylla* species with a broad distribution in the Iberian Peninsula (*H*. *spinosa*, *H*. *cochleata* subsp. *cochleata*, *H*. *lapeyrouseana*, *H*. *saxigena*, *H*. *ligustica* and *H*. *pyrenaica*). B) Additional *Hormathophylla* species which are present exclusively in the southwestern Iberian Peninsula (*H*. *cochleata* subsp. *Baetica*, *H*. *reverchonii*, *H*. *cadevalliana*, *H*. *longicaulis*, and *H*. *purpurea*).

The genus *Hormathophylla* contains about 10 species distributed in the western Mediterranean (from northern Algeria to southern France), many of which are narrow endemics from the Baetic ranges in SW Spain (Fig 1). These species can be distinguished morphologically from *Alyssum* by their white or pink flowers, lateral sepals which are small or not gibbous at all, non-appendiculated filaments and larger fruits between 5–10 mm [3]. Regarding fruit morphology, the genus includes a group of species, such as *H*. *spinosa* (L.) P.Küpfer, *H*. *cochleata* (Coss. & Durieu) P.Küpfer and *H*. *lapeyrouseana* (Jord.) P.Küpfer, which show cochleariform (spoon-shaped) fruits.

From a cytogenetic perspective, the great variability found in chromosome number (x = 7, 8, 11, 15[21,24]) in comparison to most of the related genera, which regularly bear a basic chromosome number x = 8, suggests that speciation processes have been accompanied by alterations in chromosome number through polyploidy and dysploidy mechanisms [21]. Küpfer [21], in his thorough study, offered a detailed analysis of possible scenarios of chromosome number evolution in *Hormathophylla*. He also pointed out the existence of four morphologically different groups in the genus that are in accordance with his hypotheses about the origin of different chromosome numbers: (1) a group encompassing species with the basic chromosome number of x = 7 including *H*. *reverchonii*, *H*. *cadevalliana*, and *H*. *longicaulis*, (2) a group with x = 8 including *H*. *ligustica* (as *H*. *halimifolia*), *H*. *saxigena* (as *H*. *macrocarpa*), and *H*. *spinosa*, (3) *H*. *pyrenaica* with x = 8 and (4) the group with x = 11 and 15 corresponding to *H*. *cochleata* and *H*. *lapeyrouseana*, respectively.

Apart from the morphological and chromosome variability, a unique trait observed in this genus is the ability to thrive on a wide range of substrates. Rešetnik et al. [16] underscored the importance of the soil in the diversification process of the tribe, supporting the monophyly and sister relationship between the two clades containing most of the Alysseae serpentinophytes: the so-called Bornmuellera and Clypeola clades. Species of *Hormathophylla* that are part of the Bornmuellera clade can be found growing on a wide range of soil types. They are found on siliceous substrates (*H*. *spinosa* and *H*. *purpurea* (Lag. & Rodr.) P. Küpfer), limestone (*H*. *pyrenaica* (Lapeyr.) Cullen & T.R.Dudley, *H*. *saxigena* (Jord. & Fourr.) D.A.German & Govaerts) or on a series of substrates rich in magnesium, such as gypsum (*H*. *lapeyrouseana*), dolomite (*H*. *reverchonii* (Degen & Hervier) Cullen & T.R. Dudley, *H*. *cadevalliana* (Pau) T.R.Dudley, *H*. *longicaulis* (Boiss.) Cullen & T.R.Dudley, and *H*. *lapeyrouseana*) or serpentine (*H*. *longicaulis*). The number of species in the genus that thrive on dolomites is remarkable, as this is a demanding substrate that shares common features with serpentines [25–27]. Whereas serpentinophytes have been thoroughly studied in Alysseae, in which some of the species have evolved to become heavy-metal hyperaccumulators [11,28–29], little is known about the evolution of species on alkaline soils. The combined presence of high levels of Mg [30–31] and a low proportion of Ca/Mg are the common conditions defining these alkaline habitats, a feature also present in serpentines [25–26]. In addition to soil chemical composition, another factor influencing plant adaptation to this potentially toxic substrate is their parallel occurrence in highly xeric environments, like steppes or rocky habitats [32].

The present study attempts to address several of the questions still unanswered about the evolution of this genus. First, considering that several groups in the tribe Alysseae were previously revealed to be poly- or paraphyletic, it was compelling to establish whether a comprehensive sampling supports *Hormathophylla* as a monophyletic genus. Secondly, given the previous uncertainty about the generic assignment of *H*. *purpurea* (considered by some authors as a separate monotypic genus, *Nevadensia* Rivas Mart.), it was necessary to elucidate its phylogenetic relationship to other *Hormathophylla* species. Thirdly, the degree of distinctiveness between pairs of species (e.g. *H*. *cadevalliana*/*H*. *longicaulis*, *H*. *ligustica/H*. *saxigena*) has not been previously inspected by molecular markers. Finally, to ascertain whether the great morphological variation could be related either to type of substrate or to taxonomic group, we performed a morphometric study of leaf trichomes. Despite the high homoplasy affecting trichome morphology in the tribe Alysseae [10,33], when studied at the generic level, it has been noted that several trichome types are often restricted to well-defined groups of genera within this tribe [11]. The morphology of trichomes was also succesfully used for the distinction of species and subspecies in some intricate species complexes of Alysseae [34–35].

Once the phylogenetic framework was established, a complementary approach was carried out to determine the putative association between substrate and lineages, including the study of species growing on more than one type of soil, the evolution of morphological traits, such as fruit or trichome morphology, and the chromosome evolution. Finally, we used the phylogeny to perform an analysis of biogeographic patterns presenting a spatial-temporal framework by molecular dating to identify patterns compatible with the different paleoclimatic Mediterranean events that could have been involved in diversification episodes over time. To carry out these objectives, we explored a set of regions in two cell genomic compartments. In the chloroplast DNA sequences, we used regions *trnL-F* and *ndhF* [16,33,36] and for nuclear we used the ITS region from ribosomal nuclear DNA, previously used in studies of genera related to *Hormathophylla* [11,16,37] to explore both the phylogenetic relationships among species as well as among different edaphic races or ecotypes [29,38].

## Materials and methods

### 2.1 Taxon sampling

We sampled all the species that were previously assigned to *Hormathophylla* with an emphasis on the most taxonomically controversial taxa from the Baetic ranges (Table 1). Each population sample consisted of ten individuals that were collected at a distance of at least ten metrers apart from each other. Plant material was dried in silica gel and stored at room temperature. In the case of *H*. *cochleata*, *H*. *ligustica* (Breistr.) Španiel, Al-Shehbaz, D.A.German & Marhold and *H*. *pyrenaica*, and of those species that were used as outgroups in plastid sequence analysis (*Fibigia clypeata* (L.) Medik., *Brachypus suffruticosus* (Vent.) V.I.Dorof., and *Alyssoides utriculata* (L.) Medik.), plants from preserved herbarium specimens were used. Geographic locations for sampled populations are shown in Fig 1.

**Table 1:** Sampled localities of the populations belonging to *Hormathophylla* species, used for molecular, chromosome and ploidy levels analyses. For each species population, information includes: locality name, locality code (COD), UTM coordinates (COOR), altitude, chromosome number and ploidy levels detected.

Species	Locality	COD	COOR X	Y	Altitude (m)	Published Chromosome number	Ploidy level
*H*. *longicaulis*	Boca de la Pescá	01	451464	4104054	1350		8x
	Las Cabras	02	553800	4215102	1600		4x
	Deifontes	03	453705	4131533	1300		8x(9) 4x(1)
	Dornajo	04	461357	4108398	2010	56^a^	8x
	Bacares	05	542091	4125909	1770		4x
	Freila	06	510744	4150776	1030		4x
	Gor	07	505275	4131714	1501		4x
	El Madroñal	08	315020	4052486	937		8x
*H*. *cadevalliana*	Los Morrones	09	514989	4084267	2170		4x
	Sierra de la Hinojora	10	541714	4153029	1640		8x
	Mahimón	11	579861	4170364	2000	28^a^	4x
	María	12	568781	4169902	1200		4x
	Tajos de la Parra	13	513112	4089371	1630		4x
	Prados del Rey	14	513224	4137318	1150	28^b^	4x
*H*. *reverchonii*	Sierra de la Cabrilla	15	516325	4191878	1890	28^a^	4x
*H*. *cochleata* subsp. *baetica*	Sierra de la Cabrilla	16	514097	4198034	1497	22^a^	4x
	Puerto Llano	17	503918	4184884	1800		
*H*. *cochleata* subsp. *cochleata*	Agoudal	18	265298	3544201	2550		4x
	Ameskar	19	761426	3488460	2200		
*H*. *purpurea*	Almirez	20	507795.9	4105037	2420		4x
	Los Cauchiles I	21	466692.2	4103851	2800		4x
	Los Cauchiles II	22	466345.0	4103462	2810		4x
	Loma de Dilar	23	464541.8	4102952	2710		4x
*H*. *spinosa*	Tizi-n-Tichka	24	654020	3462562	2200	32^c^	4x
	Calar de la Puebla	25	546452	4200284	1800		4x
	Deifontes	26	454427	4131870	1767		4x
	Los Morrones	27	514935	4084216	2157		4x
	Loja	28	395500	4109709	1284		4x
	Piedra Lobera	29	547795	4145847	1620		4x
	Pico Magina	30	457839	4177805	1871		4x
	Mahimón	31	578885	4170385	1330		4x
	Peña de los enamorados	32	320913	4064004	1718		4x
	Almirez	33	502913	4105781	2273	32^e^	4x
	Prados del Rey	34	513224	4137318	2084		4x
	Veleta	35	466671	4102775	2965		4x
*H*. *saxigena*	Quillan	36	434948	4742326	400		4x
*H*. *ligustica*	Trinità di Entracque	37	372776	4895132	1480–1600		
*H*. *pyrenaica*	Nohèdes	38	439085	4720179	1600–1800	32^a^	
*H*. *lapeyrouseana*	Galera	39	541827	4178976	900	30^d^	4x
	Calatayud	40	609993	4578238	650		4x
	Huete	41	524723	4449334	785		4x
	Pico Magina	42	459062	4172959	1592		4x
	Medinaceli	43	505622	4534908	808		4x
	Puerto de la Mora	44	462043	4124358	1430		4x
	Tórtoles de Esgueva	45	414317	4628259	876		4x
	Monteagudo de las Vicarias	46	568273	4582217	846		4x
	Villar Domingo García	47	559395	4450905	990		4x

Chromosome numbers (when known for the locality) were taken from the previously published studies:

^a^ = Kupfer 1974,

^b^ = Cueto & Blanca, 1986,

^c^ = Galland, 1988

^d^ = Morales et al., 1986

^e^ = Kupfer, 1972.

We also included *Cuprella homalocarpa* (Emb. & Maire) Salmerón-Sánchez, Mota & Fuertes and *Cuprella antiatlantica* (Fisch. & C.A.Mey) Salmerón-Sánchez, Mota & Fuertes (previously known as *Alyssum antiatlanticum* Emb. & Maire and *A*. *homalocarpum* (Fisch. & C.A.Mey.) Boiss., respectively) because of their uncertain generic affinities and their possible relationship to *Hormathophylla*. In this sense, the putative ascription of *C*. *antiatlantica* to *Hormathophylla* was informally suggested by Küpfer (in some annotated herbarium specimens, e.g. MA 121991) as well as by Maire, who highlighted its morphological similarity to *H*. *cochleata* and *Alyssum* sect. *Ptilotrichum* (C.A.Mey.) Hook.f. [21,39].

To assess monophyly of *Hormathophylla* and its position in the Alysseae tribe, we used data sets that contained up to 94 accessions belonging to the genus *Hormathophylla* and 120 more from other species of the Brassicaceae family, the first composed of ribosomal sequences (**DS1**) and others composed of plastid sequences (**DS2**) (in those cases where sequences corresponding respectively to *ndhF* and ITS sequences were available in GenBank). All datasets used are detailed in S1 Table. Analyzed outgroups were chosen according to previous phylogenetic studies [3,10,16,40]. Name, voucher information with herbarium abbreviations according to Thiers [41] and GenBank numbers for all the accessions used are shown in Table 2 (from *Hormathophylla* genus) and Table 3 (rest of taxa used in the studies). One more data set (**DS3**) was used that was composed of plastid sequences (*trnL-F*, *trnT-trnL* and *rpl32-trnl*) sequenced in the case of the genus *Hormathophylla* and species from related genera *Fibigia*, *Brachypus* and *Alyssoides*.

**Table 2:** Sampled material of the populations belonging to *Hormathophylla* species, used in molecular studies. For each sample, information includes: Species, voucher code, locality code (COD), Genbank accession number per region studied (NCBI codes), and plastid haplotype (TCS haplotype).

Species	Voucher code	COD	nrDNA	NCBI codes				TCS haplotype
				*trnL-F*	*rpl32-trnL*	*trnT-trnL*	*ndhF*	
*H*. *longicaulis*	8249-HUAL	01	KM033795	KM033564	KM033660	KM033468		H1
			KM033794	KM033565	KM033661	KM033469		H1
*H*. *longicaulis*	104707-MUB	02	KM033819	KM033544	KM033640	KM033448		H2
			KM033820	KM033545	KM033641	KM033449		H2
*H*. *longicaulis*	17152- HUAL	03	KM033796	KM033566	KM033662	KM033470		H3
			KM033797	KM033567	KM033663	KM033471		H3
*H*. *longicaulis*	28457-1-GDA	04	KM033793	KM033568	KM033664	KM033472	KP276166	H3
			KM033798	KM033569	KM033665	KM033473		H4
*H*. *longicaulis*	3426-HUAL	05	KM033805	KM033570	KM033666	KM033474		H5
			KM033806	KM033571	KM033667	KM033475		H5
*H*. *longicaulis*	8247-HUAL	06	KM033801	KM033572	KM033668	KM033476		H3
			KM033802	KM033573	KM033669	KM033477		H3
*H*. *longicaulis*	19762-HUAL	07	KM033799	KM033548	KM033644	KM033452		H6
			KM033800	KM033549	KM033645	KM033453		H6
*H*. *longicaulis*	52262-1-MGC	08	KM033803	KM033574	KM033670	KM033478		H7
			KM033804	KM033575	KM033671	KM033479		H7
*H*. *cadevalliana*	14587-HUAL	09	KM033809	KM033546	KM033642	KM033450		H8
			KM033810	KM033547	KM033643	KM033451		H8
*H*. *cadevalliana*	14596-HUAL	10	KM033816	KM033550	KM033646	KM033454		H9
			KM033817	KM033551	KM033647	KM033455		H10
*H*. *cadevalliana*	8248-HUAL	11	KM033813	KM033552	KM033648	KM033456		H11
			KM033814	KM033553	KM033649	KM033457		H11
*H*. *cadevalliana*	45168-1-GDA	12	KM033818	KM033554	KM033650	KM033458	KP276165	H12
			KM033815	KM033555	KM033651	KM033459		H12
*H*. *cadevalliana*	3467-HUAL	13	KM033811	KM033556	KM033652	KM033460		H13
			KM033812	KM033557	KM033653	KM033461		H14
*H*. *cadevalliana*	8244-HUAL	14	KM033807	KM033576	KM033672	KM033480		H6
			KM033808	KM033577	KM033673	KM033481		H15
*H*. *reverchonii*	43898-GDA	15	KM033821	KM033608	KM033704	KM033512	KP276164	H16
			KM033822	KM033609	KM033705	KM033513		H16
			KM033823					H16
*H*. *baetica*	45161-1-GDA	16	KM033788	KM033560	KM033656	KM033464	KP276162	H17
			KM033789	KM033561	KM033657	KM033465		H17
*H*. *baetica*	457505-MA	17	KM033787					H17
*H*. *cochleata*	303409-MA	18	KM033790					H18
*H*. *cochleata*	22506-HBG	19	KM033791	KM033562	KM033658	KM033466	KP276163	H18
			KM033792	KM033563	KM033659	KM033467		H18
*H*. *purpurea*	SN-HUAL	20	KM033730	KM033580	KM033676	KM033484	KP276171	H19
			KM033731	KM033581	KM033677	KM033485		H19
*H*. *purpurea*	753120–1 MA	21	KM033732	KM033582	KM033678	KM033486		H20
			KM033733	KM033583	KM033679	KM033487		H20
*H*. *purpurea*	408494–1 MA	22	KM033734	KM033584	KM033680	KM033488		H20
			KM033735	KM033585	KM033681	KM033489		H20
*H*. *purpurea*	468681-1-MA	23	KM033736	KM033586	KM033682	KM033490		H20
			KM033737	KM033587	KM033683	KM033491		H20
*H*. *spinosa*	SN-HUAL	24	KM033784	KM033610	KM033706	KM033514	KP276173	H21
			KM033783	KM033611	KM033707	KM033515		H21
*H*. *spinosa*	SN-HUAL	25	KM033781	KM033628	KM033724	KM033532		H22
			KM033782	KM033629	KM033725	KM033533		H22
*H*. *spinosa*	SN-HUAL	26	KM033773	KM033614	KM033710	KM033518		H23
			KM033772	KM033615	KM033711	KM033519		H23
*H*. *spinosa*	119-1-COA	27	KM033785	KM033612	KM033708	KM033516		H24
			KM033786	KM033613	KM033709	KM033517		H25
*H*. *spinosa*	59531-1-MGC	28	KM033764	KM033616	KM033712	KM033520		H26
			KM033766	KM033617	KM033713	KM033521		H26
*H*. *spinosa*	20996-1-COA	29	KM033768	KM033618	KM033714	KM033522		H27
			KM033767	KM033619	KM033715	KM033523		H27
*H*. *spinosa*	498236-1-MA	30	KM033775	KM033620	KM033716	KM033524		H28
			KM033776	KM033621	KM033717	KM033525		H28
*H*. *spinosa*	20994-1-COA	31	KM033769	KM033622	KM033718	KM033526		H29
			KM033770	KM033623	KM033719	KM033527		H29
*H*. *spinosa*	5656-1-MGC	32	KM033765	KM033624	KM033720	KM033528		H30
			KM033763	KM033625	KM033721	KM033529		H30
*H*. *spinosa*	20543-1-COA	33	KM033779	KM033630	KM033726	KM033534		H31
			KM033771	KM033631	KM033727	KM033535		H31
*H*. *spinosa*	421884-1-MA	34	KM033780	KM033626	KM033722	KM033530		H32
			KM033777	KM033627	KM033723	KM033531		H32
*H*. *spinosa*	443505-1-MA	35	KM033778	KM033632	KM033728	KM033536		H33
			KM033774	KM033633	KM033729	KM033537		H33
*H*. *saxigena*	60603-ARAN	36	KM033759	KM033578	KM033674	KM033482	KP276170	H34
			KM033758	KM033579	KM033675	KM033483		H34
*H*. *ligustica*	WU-020641	37	KM033756	KM033558	KM033654	KM033462	KP276169	H35
			KM033757	KM033559	KM033655	KM033463		H35
*H*. *pyrenaica*	BOUCHARD- 198	38	KM033760	KM033606	KM033702	KM033510	KP276172	H36
			KM033761	KM033607	KM033703	KM033511		H36
			KM033762					H36
*H*. *lapeyrouseana*	21612-HUAL	39	KM033747	KM033588	KM033684	KM033492		H37
			KM033742	KM033589	KM033685	KM033493		H37
*H*. *lapeyrouseana*	177547-MA	40	KM033748	KM033590	KM033686	KM033494		H38
			KM033749	KM033591	KM033687	KM033495		H39
*H*. *lapeyrouseana*	8246-HUAL	41	KM033739	KM033592	KM033688	KM033496		H40
			KM033738	KM033593	KM033689	KM033497		H40
*H*. *lapeyrouseana*	23943-GDA	42	KM033744	KM033594	KM033690	KM033498		H41
			KM033746	KM033595	KM033691	KM033499		H41
*H*. *lapeyrouseana*	SN-HUAL	43	KM033754	KM033596	KM033692	KM033500	KP276167	H42
			KM033755	KM033597	KM033693	KM033501		H43
*H*. *lapeyrouseana*	15813-HUAL	44	KM033745	KM033598	KM033694	KM033502	KP276168	H44
			KM033743	KM033599	KM033695	KM033503		H44
*H*. *lapeyrouseana*	37693-SALA	45	KM033752	KM033600	KM033696	KM033504		H45
			KM033753	KM033601	KM033697	KM033505		H45
*H*. *lapeyrouseana*	21602-HUAL	46	KM033740	KM033602	KM033698	KM033506		H46
			KM033741	KM033603	KM033699	KM033507		H46
*H*. *lapeyrouseana*	SN-HUAL	47	KM033750	KM033604	KM033700	KM033508		H18
			KM033751	KM033605	KM033701	KM033509		H18
*C*. *antiatlantica*	49591-MA		KM033824	KR269769			KP276175	
*C*. *homalocarpa*			KR269770	KR269768			KP276174	
*A*. *utriculata*	*Genbank, 97981-SALA		*KF022514	KM033540	KM033636	KM033444	*KF022859	UTR
				KM033541	KM033637	KM033445		UTR
*B*. *suffruticosus*	*Genbank,121275-SALA		*FM164657	KM033538	KM033634	KM033442	*KF022978	SUF
				KM033539	KM033635	KM033443		SUF
*F*. *clypeata*	*Genbank, 224944-SALA		*KF022650	KM033542	KM033638	KM033446	*KF022972	CLY1
				KM033543	KM033639	KM033447		CLY2

**Table 3:** List of sequences used from the NCBI database. Voucher information and NCBI codes for each species and studied sequence are detailed.

Species		NCBI	CODE
Taxon (synonym)	Voucher codes	nrADN	ndhF
*Acuston lunarioides* (Willd.) Raf.	Burri & Krendl, W-2000-11251	KF022652	KF022974
*Aethionema grandiflorum* Boiss. & Hohen.		DQ249867	NC_009266
*Aethionema saxatile* (L.) R. Br.		GQ284853	DQ288726
*Alliaria petiolata* (M.Bieb.) Cavara & Grande		AF283492-AF283493	DQ288727
*Alyssoides utriculata* (L.) Medik.	Gutermann 35181, HAL	KF022514	KF022859
*Alyssum alyssoides* (L.) L.	Schönswetter & Tribsch 6631, WU	KF022516	KF022863
*Alyssum aurantiacum* Boiss.	Döring, Parolly & Tolimir 697b, B 100132695	KF022522	KF022866
*Alyssum baumgartnerianum* Bornm.	Nazeri, All	KF022524	KF022867
*Alyssum canescens* D.C.	Bartholomew et al. 8657		DQ288728
*Alyssum corningii* T.R.Dudley	Faghihnia, Zangooei	KF022528	KF022871
*Alyssum cuneifolium* Ten.	Schönswetter & Tribsch 6467, WU	KF022530	-
*Alyssum dasycarpum* Stephan ex Willd.		KF022531	KF022873
*Alyssum diffusum subsp*. *garganicum* Španiel, Marhold, N.G.Passal. & Lihová	Schönswetter & Tribsch 7479, WU	KF022536	KF022877
*Alyssum doerfleri* Degen	Micevski, ZA	KF022537	KF022878
*Alyssum fastigiatum* Heywood	Harald Pauli (Vienna)	KF022573	KF022907
*Alyssum granatense* Boiss. & Reut.	Boršić, Galbany, Gutiérrez & Ortiz, ZA	KF022539	KF022880
*Alyssum harputicum* T.R.Dudley	Joharchi	KF022540	KF022881
*Alyssum hirsutum* M.Bieb.	Schönswetter & Tribsch 6978, WU	KF022541	KF022882
*Alyssum lenense* Adams		EF514610	-
*Alyssum lepidoto-stellatum* (Hausskn. & Bornm.) T.R.Dudley	Nazari	KF022545	KF022886
*Alyssum minutum* Schltdl. ex DC. (*A*. *marginatum* Steud. ex Boiss.)	Schönswetter & Tribsch 4375, WU	KF022549	KF022890
*Alyssum misirdalianum* Orcan & Binzet	Binzet, ANK	KF022552	KF022892
*Alyssum montanum* s.l.	Plazibat, ZA	KF022553	KF022893
*Alyssum niveum* T.R.Dudley	Ekim, Aytac, Dunan	KF022574	KF022908
*Alyssum orophilum* Jord. & Fourr. (*A*. *pedemontanum* Rupr.)	Gutermann 32467, herb. Gutermann	KF022575	KF022910
*Alyssum paphlagonicum* (Hausskn.) T.R.Dudley	Joharchi	KF022577	KF022911
*Alyssum persicum* Boiss.	Schönswetter & Tribsch 6984, WU	KF022578	KF022912
*Alyssum repens* Baumg.	Plazibat, ZA	KF022580	KF022914
*Alyssum repens* Baumg.	Schönswetter & Tribsch 4402, WU, ZA	KF022581	-
*Alyssum siculum* Jord.	Gutermann 35274, WU	KF022585	KF022918
*Alyssum simplex* Rudolphi	Boršić, Galbany, Gutiérrez & Ortiz, ZA	KF022587	KF022920
*Alyssum stribrnyi* Velen.	Dudley 34558, B 10 0209495	KF022596	-
*Alyssum strigosum* Banks & Sol.	Schönswetter & Tribsch 6975, WU	KF022597	KF022924
*Alyssum sulphureum* T.R.Dudley & Hub.-Mor.	Nazari	KF022601	KF022927
*Alyssum tetrastemon* Boiss.	Nazari	KF022602	KF022928
*Alyssum thymops* (Hub.-Mor. & Reese) T.R.Dudley	Nazari	KF022603	KF022929
*Alyssum turkestanicum* Regel & Schmalh.	Schönswetter & Tribsch 6608, WU	KF022533	KF022875
*Alyssum turkestanicum* Regel & Schmalh.	R. & E. Willing 172898, B 10 0323559	KF022606	KF022931
*Anelsonia eurycarpa* (A. Gray) J.F.Macbr. & Payson		DQ452059	DQ288729
*Arabidopsis lyrata* (L.) O’Kane & Al-Shehbaz		-	DQ288730
*Arabidopsis thaliana* (L.) Heynh.		ATU43225	NC_000932.1
*Arabis alpina* L.		KF022692	KF023012
*Asperuginoides axillaris* (Boiss. & Hohen.) Rauschert		EF514626	-
*Asta schaffneri* (S. Watson) O.E.Schulz		GQ424526	DQ288733
*Athysanus pusillus* (Hook.) Greene		GU246183	
*Aubrieta deltoidea* (L.) DC.		DQ249850	DQ288734
*Aubrieta parviflora* Boiss.		DQ357518	DQ288735
*Aurinia corymbosa* Griseb.	Niketić, Stevanović, Tomović, Vukojičić, BEO	KF022607	KF022932
*Aurinia gionae* (Quézel & Contandr.) Greuter & Burdet	Gutermann 35748, herb. Gutermann	KF022609	KF022935
*Aurinia leucadea* subsp. *diomedea* Brullo, De Marco & Giusso	Bogdanović, ZA	KF022610	KF022936
*Aurinia moreana* Tzanoud. & Iatroú	Horandl & Hadaček jun. & sen., W- 1998–04073	KF022616	KF022942
*Aurinia petraea* (Ard.) Schur	Dakskobler, ZA	KF022617	KF022943
*Aurinia saxatilis* (L.) Desv.	Alegro, ZA	KF022621	KF022945
*Aurinia sinuata* (L.) Griseb.	Plazibat, ZA	KF022627	KF022952
*Baimshania pulvinata* Al-Shehbaz		-	DQ288736
*Barbarea vulgaris* (L.) W.T. Aiton		AJ232915	DQ288737
*Berteroa incana* (L.) DC.	Schönswetter & Tribsch 6285, WU	KF022630	KF022955
*Berteroa mutabilis* (Vent.) DC.	Alegro & Bogdanović, ZA	KF022631	KF022956
*Berteroa obliqua* (Sm.) DC.	Gutermann 35727, herb. Gutermann	KF022633	KF022957
*Berteroa orbiculata* DC.	Miladinova, MKNH	KF022634	KF022958
*Biscutella cichoriifolia* Loisel.		KF022693	KF023013
*Biscutella didyma* L.		DQ452058	DQ288738
*Biscutella laevigata* L.		KF022694	KF023014
*Boechera laevigata* (Muhl. ex Willd.) Al-Shehbaz		EU274859	DQ288739
*Boechera platysperma* (A.Gray) Al-Shehbaz		EU274958	DQ288740
*Boechera shortii* (Fernald) Al-Shehbaz		EU274846	DQ288741
*Bornmuellera baldaccii* (Degen) Heywood	Eisenblätter & Willing 84137, B 100341181	KF022635	KF022959
*Bornmuellera cappadocica* (Willd.) Cullen & T.R.Dudley	Sorger, W- 1992–8212	KF022636	KF022960
*Bornmuellera davisii* (Hedge) Rešetnik,		GQ497875	-
*Bornmuellera dieckii* Degen	Hundosi, MKNH	KF022637	KF022961
*Bornmuellera emarginata* (Boiss.) Rešetnik	Gutermann 35073, herb. Gutermann	KF022666	KF022989
*Bornmuellera tymphaea* (Hausskn.) Hausskn.	Gutermann 35075, WU	KF022639	KF022963
*Bornmuellera* x *Leptoplax*	Gutermann 35072, herb. Gutermann	KF022641	KF022965
*Brassica incana* Ten.		KF022695	KF023015
*Brassica oleracea* L.		-	DQ288742
*Brassica villosa* Biv.	L. Scuderi, CAT	KF022696	-
*Braya rosea* (Turcz.) Bunge		AY353129	DQ288743
*Brayopsis colombiana* Al-Shehbaz		EU620283	EU718525
*Brachypus suffruticosus* (Vent.) V.I.Dorof.,	Khosravi	FM164657	KF022978
*Bunias orientalis* L.		-	DQ288744
*Cakile maritima* Scop.		KF022697	KF023016
*Camelina laxa* C.A.Mey.		-	DQ288747
*Camelina microcarpa* Andrz. ex DC.		KF022699	KF023018
*Capsella bursa-pastoris* (L.) Medik.		KJ623531.1	DQ288748
*Cardamine concatenata* (Michx.) O.Schwarz		DQ005988	AF198145
*Cardamine pulchella* (Hook.f. & Thomson) Al-Shehbaz & G.Yang			DQ288749
*Catolobus pendulus* (L.) Al-Shehbaz		FJ187876	DQ288732
*Caulanthus crassicaulis* (Torr.) S.Watson			DQ288750
*Chalcanthus renifolius* (Boiss. & Hohen.) Boiss.		GQ424528	DQ288752
*Chorispora sibirica* (L.) DC.		KF022702	KF023020
*Chorispora tenella* (Pall.) DC.		DQ357526	DQ288753
*Christolea crassifolia* Cambess.		DQ523423	DQ288754
*Clastopus vestitus* (Desv.) Boiss.	K. H. Rechinger & F. Rechinger 6461, B 100209496	KF022642/GQ424608	KF022966
*Cleome rutidosperma* DC.		DQ455802	DQ288755
*Clypeola aspera* (Grauer) Turrill		KF022643	KF022967
*Clypeola cyclodontea* Delile		EF514643	-
*Clypeola dichotoma* Boiss.	Strashafr & Reed, W- 21437	KF022644	KF022968
*Clypeola jonthlaspi* L.	Schönswetter & Tribsch 3860, WU	KF022645	KF022969
*Clypeola lappacea* Boiss.		EF514645	-
*Cochlearia danica* L.		KF022703	KF023021
*Conringia persica* Boiss.		-	DQ288756
*Cremolobus subscandens* Kuntze		EU620291	DQ288757
*Cuprella homalocarpa_1* (Fisch. & C.A.Mey.) Salmerón-Sánchez, Mota & Fuertes	Fayed et al. 1350, SHG	KF022542	KF022883
*Cuprella homalocarpa _2*(Fisch. & C.A.Mey.) Salmerón-Sánchez, Mota & Fuertes	Abdel Khalik, SHG	KF022543	KF022884
*Cusickiella quadricostata* (Rollins) Rollins		DQ452066	DQ288758
*Degenia velebitica* (Degen) Hayek	Liber, ZA	KF022646	KF022970
*Descurainia sophia* (L.) Webb ex Prantl		DQ418727	DQ288759
*Dilophia salsa* Thomson		-	DQ288761
*Dimorphocarpa wislizenii* (Engelm.) Rollins		AF137593	DQ288763
*Diptychocarpus strictus* (Fisch. ex M.Bieb.) Trautv.		DQ357535	DQ288762
*Dontostemon senilis* Maxim.			DQ288764
*Draba altaica* (C.A.Mey.) Bunge		AY134161	DQ288765
*Draba nemorosa* L.		GU202509	-
*Erysimum capitatum* (Douglas ex Hook.) Greene		DQ357540	DQ288766
*Erysimum cuspidatum* (M.Bieb.) DC.	Rešetnik, ZA	KF022706	KF023024
*Erysimum diffusum* Ehrh.	Bogdanović, ZA	KF022707	KF023025
*Euclidium syriacum* (L.) W.T.Aiton		DQ357544	DQ288767
*Eudema nubigena* subsp. *nubigena* Humb. & Bonpl.		EU620297	EU718545
*Eutrema altaicum* (C.A. Mey.) Al-Shehbaz & S.I.Warwick		DQ165365	-
*Eutrema heterophyllum* (W.W.Sm.) H.Hara		DQ165352	DQ288768
*Farsetia aegyptia* Turra	Schönswetter & Tribsch 4157, WU	KF022673	KF022995
*Farsetia linearis* Decne.		KF022674	KF022997
*Farsetia longisiliqua* Decne.		KF022675	KF022998
*Farsetia longistyla* Baker f.		KF022676	KF022999
*Farsetia robecchiana* Engl.		EF514651	-
*Farsetia stylosa* R.Br.		KF022677	KF023000
*Farsetia undulicarpa* Jonsell		EF514652	-
*Fibigia clypeata* (L.) Medik.	Bogdanović, ZA	KF022650	KF022972
*Fibigia clypeata* subsp. *eriocarpa* (DC.) Greuter	Hein 45–5, B 10 0209827	KF022651	KF022973
*Fibigia macrocarpa* (Boiss.) Boiss.	Döring, Parolly & Tolimir 1315, B 100132627	KF022654	KF022976
*Galitzkya macrocarpa* (Ikonn.-Gal.) V.V.Botschantz.	Wesche, HAL	KF022657	KF022981
*Galitzkya potaninii* (Maxim.) V.V.Botschantz.	Wesche, HAL	KF022659	KF022983
*Galitzkya spathulata* (Stephan) V.V.Botschantz.		EF514657	-
*Goldbachia laevigata* (M.Bieb.) DC.		DQ357546	DQ288771
*Glaucocarpum suffrutescens* (Rollins) Rollins		-	DQ288770.1
*Graellsia saxifragifolia* (DC.) Boiss.		-	DQ288772
*Halimolobos montanus* (Griseb.) O.E.Schulz		AF307639	DQ288773
*Heldreichia bupleurifolia* Boiss.		FN397988	-
*Heliophila* sp.			DQ288775
*Hesperidanthus jaegeri* (Rollins) Al-Shehbaz		GQ424569	DQ288751
*Hesperis dinarica* Beck		KF022708	-
*Hesperis laciniata* All. (H. visianii E.Fourn.)		KF022709	KF023026
*Hesperis matronalis* L.		DQ357547	DQ288776
*Hesperis rechingeri* F.Dvořák		KF022710	KF023027
*Hesperis* sp. nov.		-	DQ288777
*Hirschfeldia incana* (L.) Lagr.-Foss.		AY722470	DQ288778
*Hornungia petraea* (L.) Rchb.	Bogdanović & Ruščić, ZA	KF022705	KF023023
*Hornungia procumbens* (L.) Hayek			DQ288779
*Ianhedgea minutiflora* (Hook.f. & Thomson) Al-Shehbaz & O’Kane		AF137568	DQ288780
*Iberis amara* L.		AJ440311	-
*Iberis sempervirens* L.		-	DQ288781
*Idahoa scapigera* (Hook.) A.Nelson & J.F.Macbr.		-	DQ288782
*Iodanthus pinnatifidus* (Michx.) Steud.		GQ424539	DQ288784
*Ionopsidium acaule* (Desf.) DC. ex Rchb.		-	DQ288785
*Irania umbellata* (Boiss.) Hadač & Chrtek	Khosravi, Biglari	KF022656	KF022980
*Isatis lusitanica* L.		KF022711	KF023028
*Isatis microcarpa* J.Gay ex Boiss.		KF022712	KF023029
*Isatis tinctoria* L.		KF022713	KF023030
*Ladakiella klimesii* (Al-Shehbaz) D.A.German & Al-Shehbaz (*Alyssum klimesii* Al-Shehbaz)		EF514608	-
*Leavenworthia crassa* Rollins		GQ424541	DQ288787
*Leiospora eriocalyx* (Regel & Schmalh.) F.Dvořák		DQ357554	DQ288788
*Lepidium alyssoides* A.Gray		KF022714.1	DQ288789
*Lepidium draba* L.	Bogdanović, ZA	KF022715	KF023031
*Lepidotrichum uechtritzianum* (Bornm.) Velen. & Bornm.	Stohr s.n., B 10 0209493	KF022665	KF022988
*Lobularia arabica* (Boiss.) Muschl.	Abdel Khalik, SHG	KF022678	KF023001
*Lobularia canariensis* subsp. *marginata* (Webb ex Coss.) L.Borgen		EF514671	-
*Lobularia libyca* (Viv.) Meisn.	Abdel Khalik, SHG	KF022679	KF023002
*Lobularia maritima* (L.) Desv.	Bogdanović, ZA	KF022681	KF023004
*Lobularia maritima* (L.) Desv.	Schönswetter & Tribsch 7493, WU	KF022682	
*Lunaria annua* L. [ssp. pachyrrhiza (Borbás) Hayek]		KF022719	KF023035
*Lutzia cretica* (L.) Greuter & Burdet		EF514592	-
*Malcolmia africana* (L.) R.Br		AY237307	DQ288793
*Malcolmia orsiniana* subsp. *angulifolia* (Boiss. & Orph.) Stork	Rešetnik, ZA	KF022720	KF023036
*Mancoa hispida* Wedd.			DQ288794
*Maresia nana* (DC.) Batt.		KF022686	KF023006
*Mathewsia auriculata* Phil.		EU620300	EU874868
*Mathewsia foliosa* Hook. & Arn.		DQ357563	EU718555
*Matthiola farinosa* Bunge		DQ357565	DQ288796
*Matthiola fruticulosa* subsp. *fruticulosa* (L.) Maire		KF022688	KF023008
*Matthiola incana* (L.) W.T. Aiton		KF022689	KF023009
*Matthiola integrifolia* Kom.			DQ288795
*Matthiola pulchella* Tineo ex Guss.		KF022690	KF023010
*Matthiola rupestris* (Raf.) DC.		KF022691	KF023011
*Meniocus aureus* Fenzl	Misirdali, Orcan	KF022523	-
*Meniocus linifolius* (Stephan ex Willd.) DC.	Schönswetter & Tribsch 6607, WU	KF022546	KF022887
*Meniocus meniocoides* (Boiss.) Hadač & Chrtek		EF514612	-
*Menonvillea hookeri* Rollins		-	DQ288797
*Moriera spinosa* Boiss.		GQ424545	DQ288798
*Morettia philaeana* (Delile) DC.		KF022687	KF023007
*Mostacillastrum orbignyanum* (E.Fourn.) Al-Shehbaz		-	DQ288799
*Myagrum perfoliatum* L.		GQ424547	DQ288800
*Nasturtium officinale* W.T.Aiton		AY254531	DQ288801
*Neotorularia korolkowii* (Regel & Schmalh.) Hedge & J.Léonard		AY353156	DQ288803
*Neslia paniculata* (L.) Desv.		KF022700	KF023019
*Neuontobotrys elloanensis* Al-Shehbaz			DQ288802
*Neuontobotrys frutescens* (Gillies ex Hook. & Arn.) Al-Shehbaz		DQ288827	AY958595
*Neuontobotrys linearifolia* (Kuntze) Al-Shehbaz		-	DQ288821
*Nevada holmgrenii* (Rollins) N.H.Holmgren		DQ452061	DQ288829
*Thlaspi cochleariforme* DC.		DQ249838	DQ288804
*Noccaea* sp.		-	DQ288805
*Odontarrhena borzaeana* (Nyár.) D.A.German	Rešetnik, ZA	KF022525	KF022868
*Odontarrhena chalcidica* (Janka) Španiel, Al-Shehbaz, D.A.German & Marhold,	Rešetnik, ZA	KF022526	KF022869
*Odontarrhena condensata* subsp. *flexibilis* (Nyár.) Španiel, Al-Shehbaz, D.A.German & Marhold	Eren & Parolly 7550, B 100208573	KF022527	KF022870
*Odontarrhena corymbosoidea* (Formánek) Španiel, Al-Shehbaz, D.A.German & Marhold	Micevski, ZA	KF022529	KF022872
*Odontarrhena gevgelicensis* (Micevski) Španiel, Al-Shehbaz, D.A.German & Marhold	Micevski, MKNH	KF022538	KF022879
*Odontarrhena kavadarcensis* (Micevski) Španiel, Al-Shehbaz, D.A.German & Marhold,	Micevski, MKNH	KF022544	KF022885
*Odontarrhena markgrafii* (O.E.Schulz) Španiel, Al-Shehbaz, D.A.German & Marhold	Rešetnik, ZA	KF022548	KF022889
*Odontarrhena muralis* (Waldst. & Kit.) Endl.	Frajman, Schönswetter & Bardy, ZA	KF022568	KF022903
*Odontarrhena nebrodensis* (Tineo) L.Cecchi & Selvi	Brullo, CAT 041739	KF022572	KF022906
*Odontarrhena obtusifolia* (Steven ex DC.) C.A.Mey.	Gutermann 33113, WU	KF022515	KF022862
*Odontarrhena serpentina* (Micevski) Španiel, Al-Shehbaz, D.A.German & Marhold	Micevski, MKNH	KF022582	KF022915
*Odontarrhena serpyllifolia* (Desf.) Jord. & Fourr.	Plazibat, ZA	KF022583	KF022916
*Odontarrhena skopjensis* (Micevski) Španiel, Al-Shehbaz, D.A.German & Marhold	Micevski, ZA	KF022594	KF022923
*Odontarrhena tortuosa* (Waldst. & Kit. ex Willd.) C.A.Mey.	Cigić & Boršić, ZA	KF022604	KF022930
*Olimarabidopsis pumila* (Stephan) Al-Shehbaz, O’Kane & R.A. Price		DQ310528	DQ288807
*Oreoloma violaceum* Botsch.		DQ357576	DQ288808
*Parlatoria rostrata* Boiss. & Hohen.		GQ424552	DQ288809
*Peltaria alliacea* Jacq.		KF022717	KF023033
*Pennellia brachycarpa* Beilstein & Al-Shehbaz		-	DQ288811
*Pennellia longifolia* (Benth.) Rollins		AF307627	DQ288810
*Phoenicaulis cheiranthoides* Nutt.		DQ399121	DQ288812
*Phyllolepidum cyclocarpum* (Boiss.) Cecchi	Döring, Parolly & Tolimir 1110, B 100132672	KF022667	KF022990
*Phyllolepidum rupestre* (Ten.) Trinajstić	Gutermann 35182, WU	KF022669	KF022992
*Physaria floribunda* Rydb.		-	DQ288813
*Physoptychis caspica* (Hablitz) V.V.Botschantz.		KF022671	KF022994
*Planodes virginicum* (L.) Greene		GQ424554	DQ288814
*Polanisia dodecandra* (L.) DC.		DQ455816	DQ288815
*Polyctenium fremontii* (S. Watson) Greene		EU275076	DQ288816
*Pseudocamelina campylopoda* Bornm. & Gauba ex Bornm.		-	DQ288817
*Resetnikia triquetra* (DC.) Španiel, Al-Shehbaz, D.A.German & Marhold	Rešetnik, ZA	KF022655	KF022979
*Rhammatophyllum erysimoides* (Kar. & Kir.) Al-Shehbaz & O.Appel		-	DQ288818
*Romanschulzia* sp.		-	DQ288819
*Savignya parviflora* (Delile) Webb		KF022698	KF023017
*Schizopetalon biseriatum* Phil. ex Gilg & Muschl.		EU620313	EU718565
*Schizopetalon rupestre* (Barnéoud) Barnéoud ex Reiche		EU620314	EU874870
*Selenia dissecta* Torr. & A.Gray		GQ424557	DQ288822
*Shangrilaia nana* Al-Shehbaz, J.P.Yue & H.Sun		-	DQ288823
*Sisymbriopsis mollipila* (Maxim.) Botsch.		AY353157	DQ288824
*Sisymbriopsis yechengnica* (C.H.An) Al-Shehbaz, C.H.An & G.Yang		AY353161	DQ288825
*Sisymbrium altissimum* L.		AF531572	DQ288826
*Smelowskia annua* Rupr.		AY230610	DQ288831
*Smelowskia calycina* (Stephan) C.A.Mey.		DQ249836	DQ288828
*Smelowskia tibetica* (Thomson) Lipsky		DQ249858	DQ288774
*Sobolewskia caucasica* N.Busch		KF022718	KF023034
*Solms-laubachia linearis* (N.Busch) J.P.Yue, Al-Shehbaz & H.Sun		DQ523417	DQ288760
*Solms-laubachia zhongdianensis* J.P. Yue, Al-Shehbaz & H.Sun		DQ523415	DQ288830
*Stanleya pinnata* (Pursh) Britton		EU620319	DQ288832
*Stenopetalum nutans* F.Muell.			DQ288833
*Sterigmostemum acanthocarpum* (Fisch. & C.A.Mey.) Kuntze			DQ288834
*Stevenia canescens* (DC.) D.A.German		KF022716	KF023032
*Streptanthus squamiformis* Goodman		-	DQ288835
*Taphrospermum altaicum* C.A. Mey.	Bartholomew et al.8485	-	DQ288836
*Tetracme pamirica* Vassilcz.		-	DQ288837
*Thelypodium laciniatum* (Hook.) Endl.		EU620328	DQ288838
*Thlaspi arvense* L.		AF336152-AF336153	DQ288839
*Thlaspi perfoliatum* L.		KF022704	KF023022
*Turritis glabra* L.		DQ518389	DQ288840

### 2.2 DNA isolation, PCR amplification and sequencing

Plant material was desiccated (0.10–0.05 g) and ground to a fine powder with a mixer mill MM400 (Resch, Haan, Germany). Total DNA was extracted using a DNAeasy Plant Mini Kit (Qiagen), and average concentration was estimated with the aid of a spectrofluorometer (Synergy Mx Microplate Reader, Biotek).

The ribosomal region (ITS1-5.8S-ITS2) was amplified in a reaction volume of 20 μL, which contained 10 ng of genomic DNA, 1 μM each primer (C26A and N-NC18S10 [42]), 0.25 μL of Gotaq polymerase (5uds/μl) (Promega, Madison, Wisconsin, USA), 1.5 μL MgCl_2_ (25 mM), and 1.6 μL dNTPs (2.5mM). PCR conditions were: 5 min at 95 °C, followed by 35 cycles of 1 min at 95 °C, 1 min at 50 °C and 1 min at 72 °C each, followed by an incubation at 72°C for 10 min. PCR amplification products were purified using a Genelute PCR cleanup Kit (Sigma-Aldrich, St Louis, MO, USA). Sequencing reactions were performed by means of the BigDye Terminator V3.1 (Applied Biosystems, Foster City, CA, USA) sequencing kit. Sequences were purified following manufacturer recommendations, and using the same primers as in the PCR. Reaction products were run on an ABI 3100 Avant (Applied Biosystems) automatic sequencer.

For cpDNA sequences, three regions were selected, according to the degree of polymorphism shown. These sequences were: *trnL*-*trnF*, *trnT-trnL* and *rpl32-trnL*. In the phylogenetic analysis of Alysseae, a fragment of the *ndhF* region was also included [16]. Primers used in the amplification reaction were those proposed by Shaw et al. [43] (in *rpl32-trnL* fragment), Taberlet et al. [44] (in the case of *trnL-trnF* and *trnT-trnL*) and Beilstein et al. [33] (in *ndhF* fragment). PCR settings were those suggested by Shaw et al. [43,45] with minor modifications. Purification of amplification products and sequencing reactions were performed as in the ITS region. All sequences were uploaded to Genbank and accession numbers are shown in Table 2.

### 2.3 Sequence analysis and haplotype identification

Forward and reverse sequences were compared, assembled and corrected where necessary using ClustalW [46] and BioEdit V 7.0.5.3 [47], to establish a consensus sequence in each sample. In the case of ribosomal sequence alignment, the use of Muscle [48] was necessary. Following the recommendations of Fuertes-Aguilar et al. [49–50], sites were considered as polymorphic (additive polymorphic sites or APS) when they exhibited more than one nucleotide in the chromatogram, an when the weakest signal was at least 25% of the strongest one. All of the obtained sequences were submitted to NCBI GenBank.

The evolution model represented by a phylogenetic tree, does not always adequately describe evolutionary scenarios like hybrid speciation, recombination or chloroplast capture via introgression [51]. Hence, reconstruction of phylogenetic networks may help in the interpretation of relationships [51–52]. For this particular purpose, one analysis was performed with data set **DS3**, which contained concatenated sequences from the amplified plastid regions (*trnL-trnF*, *trnT-trnL* and *rpl32-trnL*), obtained from the samples of the genus *Hormathophylla*, and the outgroup from the genera *Fibigia* and *Alyssoides* Mill. (in total 96 sequences). Statistical parsimony networks (statistical parsimony algorithm [52]) were constructed using the program TCS V. 1.21 [53]. In this analysis, gaps were treated as missing data.

### 2.4 Phylogenetic analysis

Phylogenetic analyses were achieved by using Bayesian Inference (BI) and Maximum Likelihood (ML) inference. A group of data sets were created for different analyses (S1 Table). In the case of ribosomal sequences, we used data sets **DS1** and **DS4**, the first with an extended sampling to cover the whole tribe Alysseae, and the second with the sampling focused on *Hormathophylla*. In the case of chloroplast sequences, we used two data sets: **DS2** for the analysis of the whole tribe and **DS3** for the analysis centered on the genus. We also assembled a concatenated matrix with sequences belonging to ribosomal and cpDNA sequences, creating a new data set called **DS5**.

For the BI phylogenetic analysis, we employed MrBayes 3.1.2 [54–55]. Selection of the best-fit model of nucleotide substitution was carried out according to Bayesian and Akaike Information Criteria (BIC and AIC), as implemented in jModelTest [56]. 5x10^6^ generations were produced, with four Markov chains and sampling every 100^th^ generation. The first 10% of the sampled trees was discarded as burn-in. With the remaining trees, a 50% majority rule consensus tree was generated and posterior probability used as an estimation of clade support.

ML analysis was performed using RAxML 7.0.4 software [57–58]. Phylogenetic analyses were carried out through 1000 fast bootstrap analyses followed by a search for the best resulting ML tree in a single run. Because of the demanding computational requirements, a version of the program located in the web CIPRES-gateway (http://8ball.sdsc.edu:8888/cipres-web/home) was used. Following recommendations of [59], the General Time Reversible model (GTR) was used with an alpha parameter for the shape of the gamma distribution to account for among-site rate heterogeneity for both datasets. Highly congruent topologies were obtained using the parsimony ratchet [60], following the same method as in [61].

### 2.5 Data analysis for the estimation of divergence times

Two data subsets, obtained from **DS1** and **DS2** respectively, called **DS6** and **DS7** were used for the estimation of divergence times. As in the phylogenetic analyses, AIC and BIC criteria were considered to determine the substitution model that best fitted the data sequences. Three independent replicates of the BI analysis of the genus *Hormathophylla* phylogeny generated trees with similar topology. Only one run was used for the construction of the consensus tree, with 25% of sampled trees deleted as burn-in.

Molecular dating analyses were performed with BEAST v1.8 [62], a program designed to estimate divergence times by means of the Bayesian Markov chain Monte Carlo (MCMC) approach. The tool Beauti [63] was used to edit the input file for BEAST. Thereafter, a relaxed molecular clock model was implemented using the uncorrelated lognormal parametric algorithm [62]. All the analyses were carried out on the assumption of a birth-death speciation model as a tree prior [64], assuming constant rates of speciation-extinction per lineage. Four runs with two chain, were performed for the dating analysis, each with the MCMC chain length set to 75,000,000, so as to extract every 10,000th and sample every 10,000 trees. The results of the BEAST analyses were checked in Tracer 1.5 [65] for model of likelihood and parameters convergence between each run and that each run had reached a stationary state.

Both chains were combined using LogCombiner 1.8, after discarding the first 10% of the sampled trees. Results were considered reliable once effective sample size (ESS) values of all parameters were above 200 [63]. A Maximun Clade Credibility (MCC) tree was generated using TreeAnnotator 1 [63]. FigTree 1.4.0 [66] was used to display the resulting tree, including confidence intervals. Phylogenies were calibrated using two calibration points, a fossil and a secondary one. A *Thlaspi primaevum* fossil [67], dated at 30.8–29.2 Ma [68], was used as a calibration point for the split between *Alliaria petiolata* (M. Bieb.) Cavara & Grande and *Thlaspi arvense* L (prior distribution lognormal, mean/SD = 0.5/1, offset = 28). A secondary calibration point of 20.12 Ma (95% HPD: 7.46–34.42) was used in the root of the tree with the oldest common ancestor of the tribe Alysseae [12] (prior distribution normal, mean/SD = 19.3/4.3).

### 2.6 Scanning electron microcopy study. Trichome morphology

Scanning Electron Microscopy (SEM) was used to examine trichome morphology of selected taxa from the tribe Alysseae. Taxa and voucher specimens are listed in S2 Table. These studies were carried out at the Real Jardín Botánico SEM facility. Small portions of leaves were taken and mounted on a stub using double adhesive tape, and sputter-coated with gold in a Sputter Coater Balzers model SCD 004 with a thickness of about 50–700 μm. The specimens were visualized with a Hitachi S3000N digital electron microscope, operated at 25 kv. Since stellate trichomes are usually dichotomously branched up to three orders and the branch number varied depending on their order, we restricted the term ray to the last order number of ramification. This number has to be counted on the upper side of the leaves, where its presence is usually higher. Indument density and number of indument layers, as well as presence of rugosity in the epidermis, were registered and trichomes classified based on type of branching number of first-order arms, number of succesive divisions per main arm, and number of terminal rays. In *Hormathophylla* species, a series of measures were taken for the following parameters of stellate trichomes: longest and shortest ray length, and its ratio (roundness index), central disc diameter, trichome ray length between same order branching, branching angle and branch diameter.

### 2.6 Flow cytometry and estimation of DNA ploidy levels

Flow cytometry was used to measure relative nuclear DNA content and infer ploidy levels of the studied populations. Ploidy was first analyzed in plants of *Hormathophylla* from populations with known chromosome numbers [21,69–72] and then used to infer ploidy level in other populations. *Solanum lycopersicum* L. ‘Stupické polní rané’ (2C = 1.96 pg [73]) or *Bellis perennis* L. [74] were used as internal standards. Up to 10 samples per locality were analyzed when possible (Table 1).

Fresh and young leaves were dried in silica gel immediately after field collection and were stored at 25°C [75]. For the isolation of nuclei, desiccated leaves (0.5 cm^2^) were co-chopped with the fresh leaf tissues from a standard individual using a razor blade in a Petri dish with 1 mL of ice-cold Otto I buffer [76]. The resulting suspension was filtered through a 42 μm nylon mesh and incubated for at least 5 min at room temperature. One milliliter of a solution containing Otto II buffer [76] supplemented with 2-mercaptoethanol (2μL/mL) and DAPI (4μg/ml) was added to the flow-through fraction, and stained for 1–2 min. Intensity of fluorescence of 5000 particles (dyed nuclei) was measured using a Partec Cyflow ML instrument with an HBO-100 mercury arc lamp (Partec GmbH, Munster, Germany). The flow cytometry histograms were evaluated using Partec FLO MAX V. 2.4d (Partec GmbH) software. The reliability of the measurements was assessed by computing the coefficients of variation (CVs) from both the analyzed and the standard samples. All analyses above the CV threshold value of 5% were rejected.

## Results

### 3.1 Phylogenetic analysis of the genus *Hormathophylla* using ribosomal sequences

According to the results of the expanded ITS data set (S1 Fig), which included a comprehensive sampling of **DS1**, the sister group of the genus *Hormathophylla* (0.91 PP; 61% BS) was composed of *Acuston* Raf., *Alyssoides*, Mill., *Brachypus* Ledeb., *Clastopus* Bunge ex Boiss., *Degenia* Hayek, *Fibigia* Medik., *Irania* Hadač & Chrtek, *Lutzia* Gand., *Physoptychis* Boiss., *Pterygostemon* V.V. Botschantz and *Resetnikia* Španiel, Al-Shehbaz, D.A.German & Marhold, all of them grouped into a well-supported clade (0.99 PP; 79% BS). This group is related to that consisting of the *Bornmuellera* and *Phyllolepidum* genera (0.97 PP/ 74% BS), also well supported, making up the Bormuellera clade (1.00 PP; 100% BS). All of these genera include species that mostly grow on alkaline substrates (both serpentine and dolomite). The Bornmuellera clade is a sister group of the Clypeola clade (1.00 PP; 97% BS), composed of the genera *Clypeola*, *Odontarrhena* and *Meniocus* (represented by *A*. *linifolium* Stephan ex Willd). The rest of taxa described by Rešetnik et al. [16] (belonging to *Aurinia* and *Alyssum*) were present and well differentiated.

**Fig 2:**
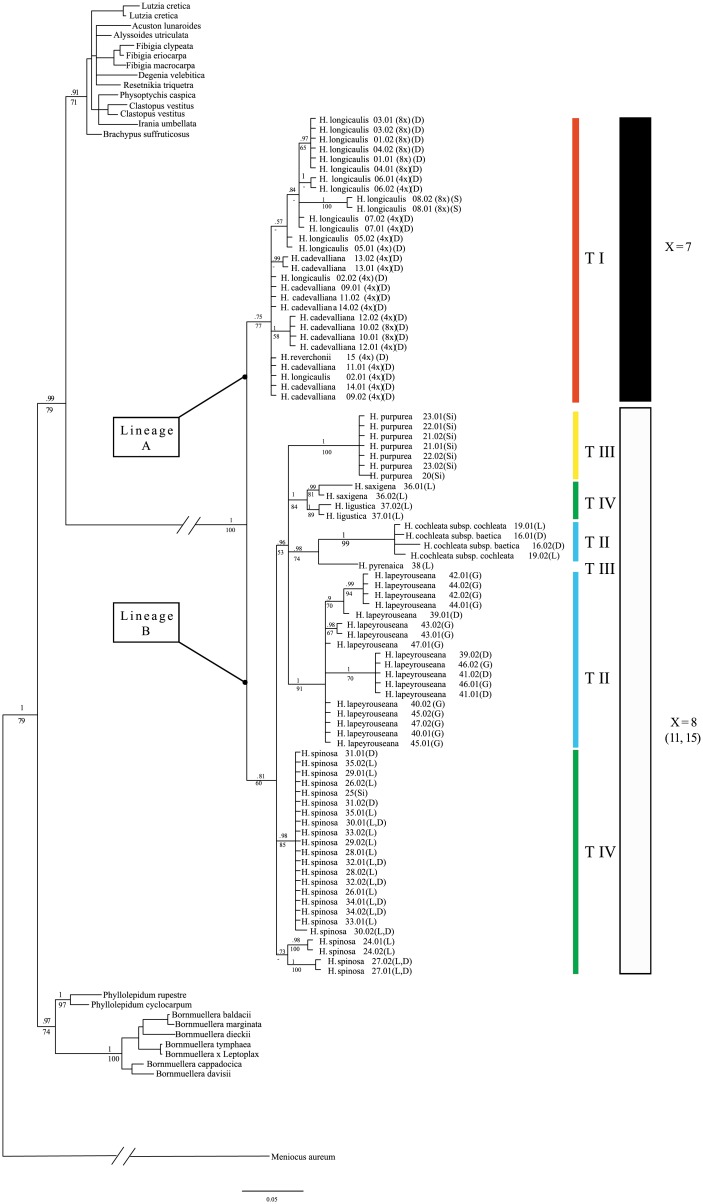
Phylogenetic relationships inferred by ML and BI analyses of the 114 samples belonging to *Hormathophylla*, based on the ribosomal sequence ITS1-5.8S-ITS2, shown as a majority rule consensus tree. Posterior probability and bootstrap support values are shown above and below the branches. Trichome types, as described in S5 Table, across different species are coded as T1, T2, T3, and T4. Basic chromosome number (x) is indicated by colored bars. Substrate type of the populations where sequences come from are coded as follows: D: dolomite; G: gypsum; L: limestone; S: serpentine; Si: siliceous.

The ITS data set (**DS4**) with an enhanced sampling of *Hormathophylla* contained 699 sites, of which 147 were variable and 68 considering only sequences from *Hormathophylla*. A total of 55 sites correspond to additive sites (S3 Table). In the jModeltest analysis, the evolutionary model was selected by both AIC and BIC (S1 Table). The tree topologies obtained in Bayesian inference and Maximum likelihood analyses were similar, although the branch support values and some connections varied slightly. The resulting trees are shown in Fig 2, centered in *Hormathophylla* species and in S1 Fig with an enlarged sampling of outrelated genera.

### 3.2 Species diversification in *Hormathophylla*

According to our ITS tree results (Fig 2), *Hormathophylla* can be considered a monophyletic genus (1.00 PP; 100% BS), which would include *H*. *cochleata* and *H*. *purpurea* as taxa belonging to this group. The split between two lineages, hereafter called A and B, is clear. Both lineages correspond to the two groups of species previously detected based on basic chromosome numbers and are well supported (lineage A was 0.75 PP; 77% BS and lineage B 0.81 PP; 60% BS).

Lineage A (Fig 2) is composed of *H*. *longicaulis*, *H*. *cadevalliana* and *H*. *reverchonii*. Within lineage A, no clear differentiation between species is observed, although it is supported for most of the populations from *H*. *longicaulis* (0.57 PP;—BS). Populations belonging to *H*. *cadevalliana* appear merged with the other two, sometimes grouped into a clade, as is the case of populations 12 and 10 (1.00 PP/ 58% BS). In lineage B (Fig 2), two subclades encompass all of the populations of *H*. *spinosa*, one containing populations 24 and 27 (0.73 PP; -- % BS) and the rest in a second one (0.98 PP; 85% BS). The third clade in this trichotomy contains the rest of the species (0.96 PP; 53% BS). Among them, *H*. *purpurea* (1.00 PP; 100% BS) and *H*. *lapeyrouseana* (1.00 PP; 91% BS) remain well differentiated. *H*. *ligustica* and *H*. *saxigena* form a well-defined clade (1.00 PP; 84% BS). The two *H*. *cochleata* subspecies, *baetica* and *cochleata*, group into a well-supported clade with respect to the rest of the lineage B species (1.00 PP; 99% BS) grouped into a clade along with *H*. *pyrenaica* (0.98 PP; 74% BS). There is some within-species, and even intrapopulational, variation; for instance, in *H*. *lapeyrouseana*, populations 39, 41 and 46 are well separated from the rest (1.00 PP; 70% BS), whereas the other branch contains individuals from the southernmost populations 39, 42 and 44 (0.9 PP; 70% BS).

### 3.3. Haplotype diversity in *Hormathophylla*

The plastid DNA sequences obtained both in *Hormathophylla* and the outgroups ranged between 477–484, 829–850, 892–854 bp for *trnL-trnF*, *trnQ-rpl16* y *trnT-trnL*, respectively. The combined matrix of the three regions, DS3 (Table 2) (2.485 bp), showed 131 variable sites and 40 indels (S4 Table). The combined matrix generated 52 haplotypes (Table 2) distributed as follows. Whereas some species like *H*. *reverchonii* (H16), *H*. *cochleata* (H17, H18), *H*. *ligustica* (H35), *H*. *saxigena* (H34), *H*. *pyrenaica* (H36), and *H*. *purpurea* (H19, H20) displayed one or two haplotypes per species, other species exhibit a richer nucleotide variation in the chloroplast regions analyzed. For example, *H*. *lapeyrouseana* shows 11 haplotypes, one of them shared with *H*. *cochleata* (H18), plus ten more exclusive (H37-H46). In *H*. *spinosa*, were found 13 haplotypes (H21-H33), most of them restricted to one population. In the case of *H*. *cadevalliana*, which presents nine haplotypes (H6, H8-H15), one of them (H6) was shared by *H*. *longicaulis*, where six more haplotypes were found (H1-H7).

**Fig 3:**
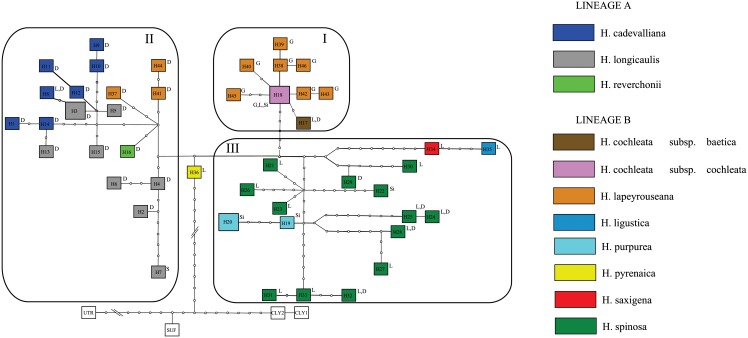
Statistical parsimony network based on plastid sequences (*trnL-trnF*, *trnT-trnL* and *rpl32-trnL*), for species of *Hormathophylla*, and *Alyssoides*, *Brachypus* and *Fibigia*, as outgroups. Lineages obtained in BI trees are represented. Haplotypes are grouped into three groups I, II and III. The substrate type of the populations where the haplotypes occur is coded as follows: D: dolomite; G: gypsum; L: limestone; S: serpentine; Si: siliceous.

The internal relationships among the chloroplast haplotypes based on the same haplotype data set (DS3, Table 2) were resolved into one single TCS network (Fig 3). Among the 96 analyzed sequences, we found 50 haplotypes, including those in the outgroup (one for *Fibigia suffruticosa* and *Alyssoides utriculata* and two for *Fibigia clypeata*). We found *H*. *pyrenaica* to be the species with haplotypes closest to those present in outgroups, separated by 165 steps from the nearest outgroup. The rest of the species were split into three well-differentiated haplotype groups. The first one (haplogroup I) was composed of haplotypes belonging to the species *H*. *cochleata* and *H*. *lapeyrouseana*, which are placed in the ITS lineage B. This haplogroup was separated by seven steps to the nearest taxon (*H*. *pyrenaica*). *H*. *cochleata* and some populations of *H*. *lapeyrouseana* shared the same haplotype. The longest distance between haplotypes within this group was of five mutational steps. The most frequent haplotype was present in five sequences, three populations and two subspecies (*H*. *cochleata* subsp. *cochleata* and *H*. *cochleata* subsp. *baetica*). A second group (haplogroup II) was composed of haplotypes from species belonging to ITS lineage A, along with several individuals from the southernmost populations of *H*. *lapeyrouseana*. They were distanced three steps from the nearest species of the neighbor group, *H*. *pyrenaica*. In this group, most haplotypes correspond to *H*. *longicaulis*, which accumulates up to 7 haplotypes separated into two subgroups. Nine haplotypes are present in *H*. *cadevalliana*, one of which is also shared by *H*. *longicaulis*. They were separated by 19 steps. The most frequent haplotype (H3) in haplogroup II was present in four individuals, three populations and only one species. A third group (haplogroup III) consisted of haplotypes belonging exclusively to lineage B. The haplotypes belonging to *H*. *spinosa*, were tightly related to *H*. *ligustica*, *H*. *saxigena* and *H*. *purpurea*. The closest haplotype from another group was eight steps away (between *H*. *pyrenaica* and *H*. *spinosa*). Considering only haplotypes from *H*. *spinosa*, the longest distance between haplotypes was 26 steps (H27 and H30). In this haplogroup the most frequent haplotype was H20, which was present in three of the populations from *H*. *purpurea*, and separated by six steps from the nearest haplotype of *H*. *spinosa*. Regarding *H*. *saxigena*, its haplotype was separated four steps from the exclusive *H*. *ligustica* haplotype and 11 steps from nearest haplotype of *H*. *spinosa*.

There is an association between the haplotypes and the type of substrate in which the different populations grow. This association is, in some cases, independent of the taxonomic ascription. Within haplogroup I, we find *H*. *lapeyrouseana*, with most of the haplotypic lineages present on gypsum, and the two subspecies of *H*. *cochleata*, which are present on calcareous outcrops (limestone and dolomite). Within haplogroup II, we find species that were always present on alkaline soils, with a particular predominance of dolomitic or calcodolomitic substrates (*H*. *reverchonii*, *H*. *longicaulis*, *H*. *cadevalliana* and *H*. *lapeyrouseana*), but all the individuals analyzed from populations living on serpentines (*H*. *longicaulis*) are in this group. Finally, in haplogroup III, the most diverse, we find species on different substrate affinities, although most of them were predominantly on limestone (*H*. *pyrenaica*, *H*. *ligustica*, *H*. *saxigena* and *H*. *spinosa*). Moreover, *H*. *spinosa* and *H*. *purpurea* were found on siliceous substrates.

### 3.4 Within *Hormathophylla* plastid phylogenetic analysis

**Fig 4:**
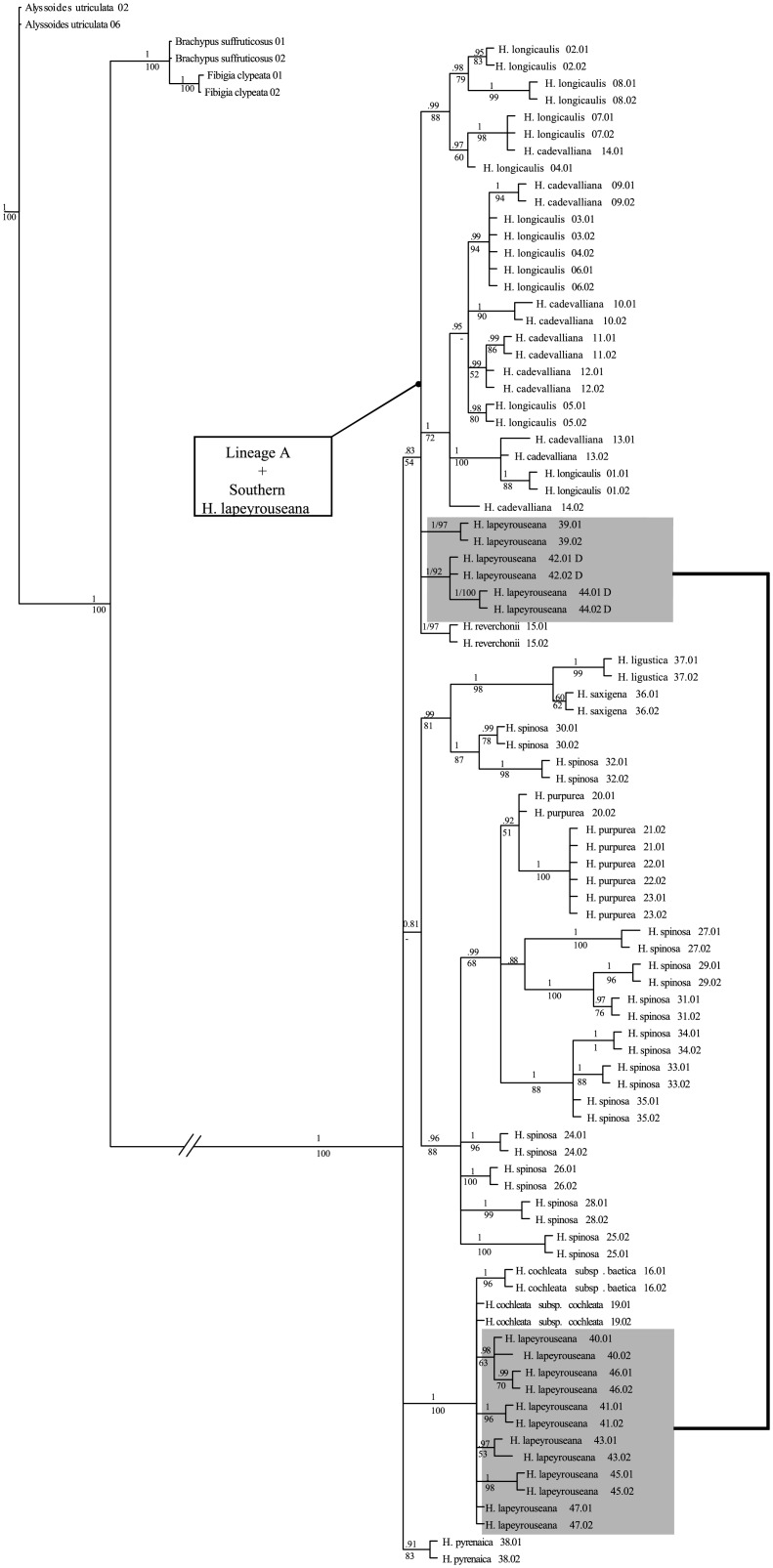
Phylogenetic relationships inferred through ML and BI analyses of the 96 samples belonging to the genus *Hormathophylla*, and the species *Fibigia clypeata*, *Brachypus suffruticosus*, and *Alyssoides utriculata*, of the chloroplast regions *trnL-trnF*, *trnT-trnL* and *rpl32-trnL*, shown as a majority rule consensus tree. Posterior probability and bootstrap support values are shown over and below the branches, respectively.

When the plastid data set (DS3) is used to infer the phylogenetic relationships within *Hormathophylla*, the resulting tree (Fig 4) exhibits a lower resolution than the topology obtained from ITS ribosomal sequences. However, the topology does not exactly match the two lineages A and B, as identified in the ITS phylogeny (Fig 2). The species included in ITS lineage A grouped into its own clade (0.7 PP; 76% BS) in the plastid phylogeny. The clade is completed with the sequences belonging to the southernmost populations of *H*. *lapeyrouseana*. Within this clade several sublineages are identified. The first corresponded to *H*. *reverchonii* individuals (1.00 PP; 97% BS), two more contained sequences from the southernmost populations of *H*. *lapeyrouseana* (for population 39, 1.00 PP; 97% BS, and for populations 42 and 44 1.00 PP; 92% BS), and two more (0.99 PP; 88% BS and 1.00 PP; 72% BS) contained samples belonging to *H*. *cadevalliana* and *H*. *longicaulis*, with many of their sequences grouped as mixtures from both species (0.97 PP; 60% BS).

The remaining species are grouped into three additional clades. A first clade comprises haplotypes from *H*. *spinosa*, *H*. *saxigena*, *H*. *ligustica* and *H*. *purpurea* (0.81 PP;—BS), and contained two subclades. The first (0.99 PP/81% BS) contained *H*. *saxigena*, *H*. *ligustica*, (1.00 PP/98% BS) and populations 30 and 32 of *H*. *spinosa* (1.00 PP/87% BS). The second contained the rest of the haplotypes from *H*. *spinosa* and *H*. *purpurea* (0.96 PP/88% BS). Nested within it, lineages from *H*. *purpurea* (0.99 PP/51 BS) make up a subclade with *H*. *spinosa* populations 27, 29, 31, 33, 34, and 35 (0.99 PP/68% BS). A third clade contained the rest of the sequences belonging to *H*. *lapeyrouseana*, *H*. *cochleata* subsp. *baetica* and *H*. *cochleata* subsp. *cochleata* (1.00 PP/100% BS). A fourth clade contained the samples belonging to *H*. *pyrenaica* (0.91 PP/83% BS).

### 3.5 Phylogenetic analysis of tribe Alysseae based on the plastid region

The analysis based on data set 3 (DS3, Table 2), which was composed of *ndhF* plastid sequences (S2 Fig), revealed similar relationships between the main lineages of *Hormathophylla* and those previously resulting from the more variable three chloroplast regions (*trnL-trnF*, *trnQ-rpl16*, *trnT-trnL*) of the *Hormathophylla* genus sampling. It was possible to detect certain differentiation of species belonging to lineage A with respect to the rest except in the case of *H*. *lapeyrouseana*. The southernmost localities of this species were tightly linked to those belonging to lineage A, making up a consistent phylogenetic group (1.00 PP; 100% BS). The relative position of *Hormathophylla* within the tribe appears to be clearly nested within a highly supported lineage (1.00 PP; 100% BS) in the clade composed of *Hormathophylla* (1.00 PP; 100% BS), *Fibigia*, *Irania*, *Clastopus* and *Degenia* among others. With a high support (1.00 PP; 83% BS), this group revealed a sister relationship with another group made up of the genus *Phyllolepidum*. The large clade also includes the genus *Bornmuellera* (1.00 PP; 100% BS).

### 3.6 Phylogenetic analysis of the combined matrix with nrDNA and cpDNA

The analysis of the concatenated sequences of ribosomal and plastid DNA considering the partitioned data (**DS5**) generated the consensus tree shown in S3 Fig. First, the monophyly of the *Hormathophylla* genus was conserved (1.00 PP), within the Bornmuellera clade (1.00 PP). As in the plastid analysis, it was not possible to differentiate species from each of the two ITS groups. Nonetheless, we found that species from lineage A came together in a well supported group. Concerning *Cuprella antiatlantica* and *C*. *homalocarpa*, these species grouped into a lineage nested within the Bornmuellera clade, as a sister group of the genus *Bornmuellera*. The phylogenetic tree was similar to that obtained from chloroplast sequences, except in the case of the relationships between the Alyssum, Clypeola and Aurinia clades.

### 3.7 Estimation of divergence times

**Fig 5:**
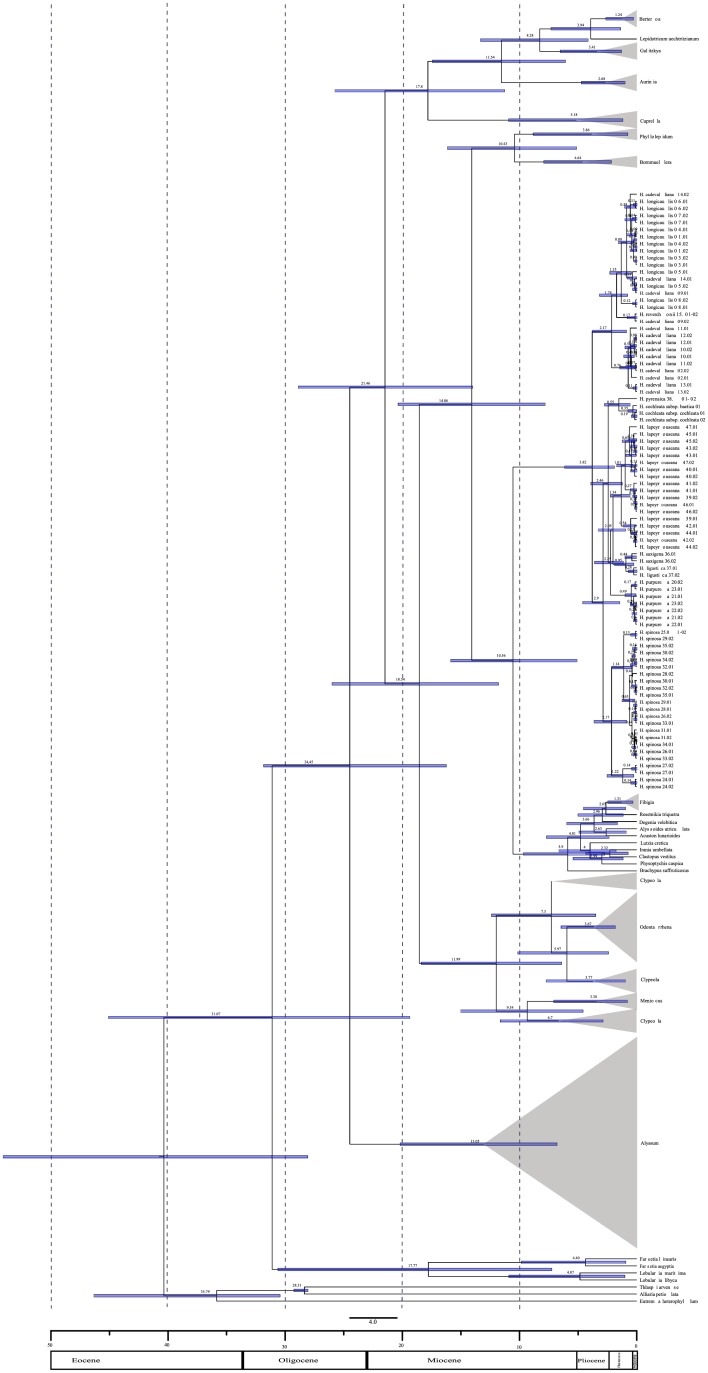
Tree generated in estimation of divergence times with BEAST using nuclear ribosomal ITS sequences. Time scale is shown in millions of years. Error bars show 95% HPD in each node. Values above each node show median with respect to probability in millions of years.

The dated phylogeny, with the estimation of divergence times (Fig 5 and S4 Fig), depending on the genome compartment used in its inference, was based on ribosomal (DS6) or plastid sequences (DS7).

As a result of the parametric analysis of the ribosomal sequences, the split between *Hormathophylla* and its most related group composed of species from the *Fibigia* genus indicated a mean age of 10.55 Ma (5.11–15.86 Ma 95% HPD), falling within in the Tortonian Age of the Late Miocene, whereas the MRCA of the genus *Hormathophylla* showed a mean age of 3.82 Ma (1.94–6.15 Ma 95% HPD), in the Zanclean Age of the Pliocene. On the other hand, the MRCA of the lineage A species was established with a mean age of 2.17 Ma (0.88–3.8 Ma 95% HPD), while for lineage B it was 2.9 Ma (1.47–4.61 Ma 95% HPD), during the Mediterranean Climate onset in the Pliocene.

The data phylogeny based on the use of plastid sequences varied widely with respect to those obtained with ribosomal sequences. Lineage A did not split well from lineage B, but if we consider the southernmost population of *H*. *lapeyrouseana*, this group split from the rest of the species 1.94 Ma (0.89–3.37 Ma 95% HPD) ago. The most recent common ancestor of the *Hormathophylla* genus was established with a mean age of 4.18 Ma (2.29–6.68 Ma 95% HPD). The split between *Hormathophylla* and the related group composed of species from the *Fibigia* genus showed a mean age of 16.14 Ma (9.70–22.88 95% HPD). The split of the Alysseae tribe with respect to the rest of the Brassicaceae was about 35.89 Ma (28.03–44.31Ma 95% HPD).

### 3.8 Leaf trichome morphology

**Fig 6:**
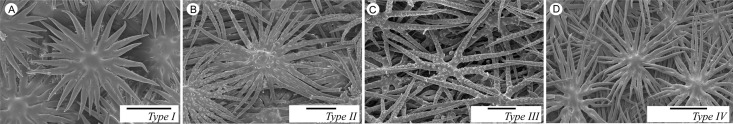
SEM photographs illustrating variation of leaf trichome morphological types in *Hormathophylla*. . A) Type I: *H*. *cadevalliana*. B) Type II: *H*. *lapeyrouseana*. C) Type III: *H*. *purpurea*. D) Type IV: *H*. *ligustica*. Scale bar = 100 μm.

The extraordinary trichome complexity and variability of the leaf blade indumentum required an additional effort of classification. All species from *Hormathophylla* presented stellate trichomes, peltate (briefly stalked), appresed and with a circular to elliptic outline ranging from 0.3 to 0.5 mm in diameter. Trichomes usually show a more or less protuberant central disc, from which a number of primary and secondary dichotomous branches extend usually in the same plane. Primary rays (four to eight) diverge from a central disc that in most species are reduced to a small central area (e.g. *H*. *purpurea* and *H*. *cochleata*). The number of last-order branches varies between 13 and 36. The central disc can show tuberculate and irregularly bulged ornamentation, but can also be smooth. All trichomes showed rounded tubercles with a different degree of density and size, that become sparser or absent at the branch tips. We were able to distinguish four types of trichomes among all species, which are present in different species depending on their phylogenetic position (Fig 6, S5 Table).

When we mapped trichome types along the phylogentic trees we found an association between lineages and the morphological types. In ITS lineage A (Fig 2), we detected a single type of trichome (type I). In this type, trichomes were stellate and broadly peltate, with an almost perfectly circular outline (diameter: 0.25–0.35 mm); they were largely overlapping and arranged in more than three layers, so dense that no leaf epidermis could be seen unless the hair cover was entirely removed. Primary rays (7–8) diverge from a rounded and undivided central disc (ca. 2/10 diameter) and are reduced to a brief, stout base bearing a total of 28–32 branch tips, almost regularly dichotomous. Rays were sparsely covered by tuberculate protrusions, that become sparser and disappear at the branch tips. These type I trichomes have not been observed in any other member of the Alysseae tribe.

With respect to ITS lineage B (Fig 2), on the contrary than in lineage A, we observed trichome differences between the species nested there. Trichomes were very dense, stellate and broadly peltated, usually with a rounded outline, and variable diameter from 0.3 to 0.4 mm. We found that primary branches were longer than those in lineage A, and tubercles were larger, more prominent and thicker, with secondary branching occurring farther from the center. Within this lineage, we could distinguish three trichome types classified depending on diameter, number of rays, presence of membranaceous structures among branches, and central disc diameter. Type II is present in *H*. *lapeyrouseana*, and the two subspecies of *H*. *cochleata*. The diameter and central disc were larger than the rest of the species (0.41 to 0.44 mm and 0.7 to 0.9 mm, respectively), with branches making up an almost circular outline, and the presence of membranaceous structures among them. Type III appears in *H*. *purpurea* and *H*. *pyrenaica*. This type of trichomes is similar to the types found in other Alysseae genera, such as *Alyssum* or *Clypeola*. Trichomes showed a low number of tubercles, lower number of branch tips (12 to 14), and an elongated central disc, with branches limited by an elliptic outline. Type IV is detected in *H*. *pyrenaica*, *H*. *spinosa*, *H*. *saxigena* and *H*. *ligustica*. In this type, trichomes showed an almost perfectly circular outline (diameter: 0.30–0.35 mm); they were largely overlapping and arranged in more than three layers, and so dense that no leaf epidermis could be seen unless the hair cover was entirely removed. Primary rays (4–7) diverge from a rounded and undivided central disc (ca. 1.5-2/10 diameter) and are reduced to a short, stout base bearing a total of 30–36 branch tips, almost regularly dichotomous. Rays were densely covered by rounded tubercles, even at the branch tips.

### 3.9 Chromosome variation and ploidy level in *Hormathophylla*

For most of the species we detected the same ploidy level (tetraploid) in most of the studied specimens (up to 10 individuals per population; Table 1). We found that all analyzed individuals from *H*. *purpurea*, *H*. *spinosa*, *H*. *cochleata*, *H*. *reverchonii* and *H*. *saxigena* exhibited a constant level of ploidy in all studied populations. We would like to note that in the particular case of samples belonging to the Morocco populations of the species *H*. *spinosa*, we did not find 2x individuals, even though they had been reported previously [77]. The occurrence of two different levels of ploidy in the same species has been recorded, in the cases of *H*. *cadevalliana* and *H*. *longicaulis*, not associated with any geographic pattern. Octoploid individuals (8x) were found in populations 1, 3 and 4 from *H*. *longicaulis* and 13 from *H*. *cadevalliana*. For these species, tetraploid individuals (4x) were found in populations 3, 5, 6, 7, and 8 in *H*. *longicaulis* and 9, 10, 11 and 12 in *H*. *cadevalliana*, which means that in some populations the two levels of ploidy co-occur. For example, in the *H*. *longicaulis* population 3, we found nine octoploid and one tetraploid individual. In both species, this is the first time that 8x level is reported for *H*. *cadevalliana*, and the first time 4x level is reported for *H*. *longicaulis*.

## Discussion

### 4.1 Origin and biogeography of *Hormathophylla*

Our results indicate that the Hormathophylla clade split from its sister group, composed by species from the genus *Fibigia*, in the Late Miocene (10.5 Ma) (Fig 5). During this period, the existence of a northern Mediterranean land corridor connecting the seas of Tethys and Paratethys [77–80] favored dispersal of a number of lineages from east to west throughout the Mediterranean region. These lineages underwent progressive isolation developing east-west disjunctions, because of the interruption of the corridor by marine transgressions in Late Miocene and the structuration of the Alps (10 Ma) [81]. Such an east-west disjunct distribution within the Bornmuellera clade is observed between lineages present in western-Mediterranean and eastern-Mediterranean, sometimes extended in central Asia (S5 Fig). This is a biogeographic pattern described in insects as the so called Kiermack disjunctions [82] but also in Mediterranean plants like in the tribe *Anthemidae* [83] and the genus *Jasione* L. [84].

Therefore, the *Hormathophylla* ancestor established in western Mediterranean diversified into two different lineages in the upper Pliocene (about 3.8–4.18 Ma ago depending on whether we consider ITS or plastid sequences). However, it is not until the transition between the Late Pliocene and Gelasian in the early Pleistocene (2.2 and 2.9 Ma for lineages A and B, respectively), during the onset of the Mediterranean climate (3.4–2.9 Ma), when the rapid diversification of *Hormathophylla* species took place, fundamentally in the Baetic ranges. This general pattern of rapid Baetic diversification in plant taxa is well documented by both paleobotanical [85–89] and molecular studies [90]. The onset of the mediterranean climate was a significant environmental change caused by the establishment of rainy temperate seasons and drought in summer months. Based on dated phylogenies of several plant genera, e.g. *Dianthus* L. [91] or *Cistus* L. [92], there was an increase in rates of diversification with the establishment of the Mediterranean climate with dry summers.

The small area of distribution of most of the species from the genus, (*H*. *reverchonii*, *H*. *cochleata* subsp. *baetica*, *H*. *purpurea* or *H*. *pyrenaica*), all of them with a Pleistocene crown age, would additionally suggest that fragmenting patterns have played an important role in the biogeographical diversification of *Hormathophylla*. The influence of the Pleistocene stadial-interstadial dynamics in the southern Europe ranges has already been described in other genera like *Armeria* Willd. and *Anthyllis* L. [93–96].

The consequences of speciation by geographical isolation affected mostly in Baetic ranges (as well as in northern Morocco and southern France), followed by secondary contacts, with expansion and contraction of species distribution reflecting climatic fluctuations of the Quaternary period [97–98]. During the glacial period, broad areas at middle altitudes were covered by cold steppe and tundra biomes, providing suitable habitats for mountain plants [99]. It is possible that interglacial warming caused populations to retract to the top of the mountains [94,100]. The glacial/interglacial cycles in southern Iberian ranges helped to shape the present distribution of genetic lineages, which are geographically contiguous with each species adapting to a different substrate [101–103].

### 4.2 The genus *Hormathophylla* is monophyletic

Our analyses confirm the monophyly of *Hormathophylla*, resolving two conflicting questions regarding the limits of the genus: the separation of *H*. *purpurea* as a monotypic genus and the generic ascription of *Alyssum antiatlanticum* to *Hormathophylla*. *H*. *purpurea* had been proposed as a separate monotypic genus, *Nevadensia* (*Nevadensia purpurea* (Lag. and Rodr.) Rivas Mart.) [22] based on the morphological and ecological differences existing between *H*. *purpurea* and the remaining species of *Hormathophylla*. This view has been followed in some recent publications [104]. However, Küpfer [21] who initially considered that *Ptilotrichum purpureum* (Lag. & Rodr.) Boiss. should be treated as *Alyssum purpureum* Lag. & Rodr., later transfered this species to *Hormathophylla* due to the color of flowers (pink), unusual in *Alyssum* [18]. Our plastid phylogenetic analyses not only confirm Küpfer’s view, but also reveal a close relationship of this species with *H*. *spinosa*, the only other species with pink petals (Fig 4). The ITS phylogeny reveals the placement of this species in lineage B, and is totally congruent with its basic chromosome number x = 8 detected by Contandriopoulos [21].

The second potentially disruptive element in the monophyly of *Hormathophylla* was *Alyssum antiatlanticum*. *A*. *antiatlanticum* is an isolated species in the genus that Maire [39] assigned to *Alyssum* sect. *Psilonema* (C.A.Mey.) Hook. f. because of its elongated cylindrical nectaries. The putative assignment of *A*. *antiatlanticum* to *Hormathophylla* was informally proposed by Küpfer based on some annotated specimens (e.g., MA 121991), as well as by Maire who highlighted its morphological proximity to *H*. *cochleata* (Coss. & Durieu) P.Küpfer and *Alyssum* sect. *Ptilotrichum* (C.A.Mey.) Hook.f. [21,39]. Our phylogenetic analyses constitute the genetic basis for the recently published genus *Cuprella*, which includes *C*. *antiatlantica* and *C*. *homalocarpa* and reject its phylogenetic placement within *Hormathophylla* [17].

The obtained phylogenies reveal the early split of the two lineages according to its basic chromosome number, distinguishing those belonging to lineage A, derived from x = 7, from those belonging to lineage B, probably derived from x = 7 and x = 8 via hybridization and aneuploidy [21]. This differentiation is also supported by a separation in trichome morphology. Whereas species belonging in lineage A (Type I) were very similar, species in lineage B encompass three types (II, III and IV) (S5 Table). Regarding the basic chromosome number x = 8, which is by far the most common in the tribe outside of *Hormathophylla* (171 vs 7 species), we postulate that species grouped in lineage A with 7 as a basic chromosome number are derived from a common ancestor with x = 8.

### 4.3 Relationships between *H*. *cadevalliana* and *H*. *longicaulis*

Lineage A was composed of *H*. *reverchonii*, *H*. *cadevalliana* and *H*. *longicaulis*. The low phylogenetic divergence, reflected in the low resolution between species belonging to lineage A, is supported by the almost uniform trichome type I (Fig 5) morphology observed, where no distinction in trichome types is found between *H*. *cadevalliana* and *H*. *longicaulis*, and the indumentum of both species is very similar to that of *H*. *reverchonii* (S5 Table).

An explanation for the lack of differentiation in ITS topology might suggest the occurrence of introgression among some of the taxa. In the case of *H*. *cadevalliana* and *H*. *longicaulis*, ITS sequences obtained appear intermixed in the tree (Figs 2 and 4) with the presence of additivity patterns (S3 Table). All the evidence seems to indicate that there is either a past or an ongoing hybridization process between the two species in part of their overlapping areas of distribution. Such an admixture pattern is more evident in some of the southern ranges (Baza and Filabres) where populations from both species are even sympatric. The diversity observed in ploidy level remarkably only appears in those localities where sympatric populations from two species occur, e.g. *H*. *longicaulis* population 3 groups individuals with 4x- and 8x- levels of ploidy. Likewise, other *H*. *cadevalliana* populations (14) exhibit a similar additive pattern with *H*. *reverchonii* (S3 Table). In addition, plastid haplotypes from all three species in this lineage are all clustered in the haplotype network (Fig 3). We could not find any correlation between morphological variation and level of ploidy within the same species, which would preclude the possible taxonomic recognition of infraspecific taxa based on that criterion.

If introgression between the two species is actually occurring, the lack of resolution observed in the ITS phylogenetic tree could be explained by the inclusion of sequences in a process of incomplete concerted evolution. The occurrence of individuals with ITS sequences rich in additive polymorphic sites [50] can also be favored by the presence of early generation hybrid individuals [49], in which concerted evolution of multi-copy genes is retarded by a lack of homologous loci recombination. Remarkably, in the genus *Hormathophylla*, only in *H*. *longicaulis* and *H*. *cadevalliana* has the presence of octoploid individuals been demonstrated by chromosome counts and ploidy estimations via genome size [21] (Table 1, this study). A plausible scenario in which hybridization could arise was along the succesive cycles of expansion and contraction due to cycles of glaciation that could favor the hybridization processes as previously demonstrated for other plant groups in the same soutwestern Iberian ranges [102–103].

### 4.4 Fruit morphology. Genetic differentiation between northern and southern populations in *H*. *lapeyrouseana* and in relation to adaptation to dolomitic substrates

Not many morphological traits support the species ITS lineages identified in *Hormathophylla*. All species with cochlear fruit were present within lineage B, although they could have originated separately twice: in *H*. *spinosa* and *H*. *cochleata* subsp. *cochleata*, *H*. *cochleata* subsp. *baetica* and *H*. *lapeyrouseana* clade, respectively (Fig 2). This result is in line with the considerations made by Al-Shehbaz et al. [2] in relation to the use of the fruit morphology as a taxonomical character in the family Brassicaceae. This author suggests that important changes in fruit morphology can occur faster and independently of other characters, subject to a considerable convergence and sometimes taxonomically irreproducible. There are many studies that suggest that differences of only a few genes can cause substantial variations in shape, size and dehiscence of the fruit in the family Brassicaceae, e.g. in *Arabidopsis*, genes involved in formation of fruit (FRUITFUL, MADS-box, SHATTERPROOF) can modify shape (e. g., ratio length/width) and dehiscence type [105–108].

*H*. *cochleata* subsp. *cochleata*, *H*. *cochleata* subsp. *baetica* and *H*. *lapeyrouseana* constitute a well supported group both with nuclear and plastid markers (with the exception of the southernmost localities of *H*. *lapeyrouseana*). Differentiation between *H*. *cochleata* subsp. *cochleata* and *H*. *cochleata* subsp. *baetica* has been subjected to taxonomic studies, being considered as the same [21] or vicariant species [109]. Our results support a synthetic vision, as we will consider later. Furthermore, molecular data establish a close relationship between these species and *H*. *lapeyrouseana*. This is based on the similarity of plastid haplotypes that could be caused by a process of recent hybridization, or to an incomplete lineage sorting, given the great differences that exist between their basic chromosome numbers.

On the other hand, the plastid lineage of the southernmost populations was more related to species belonging to lineage A than lineage B (within haplogroup II). This could be due to an incomplete lineage sorting in plastid sequences, as there is no clear evidence of ancestral hybridization (between species from lineages A and B, proposed by Küpfer [21]), as there are no ribosomal additivities shared. Nonetheless, this should not be ruled out, nor should the existence of horizontal chloroplast transference (haplogroup II, with a high number of plants present on dolomite), which could be associated somewhat to adaptation to special substrates.

Other existing relationships, according to the phylogeny obtained through plastid DNA, are between *H*. *spinosa* and *H*. *purpurea*, *H*. *ligustica* and *H*. *saxigena*, all with the same chromosome number. This finding could indicate the existence of a common ancestor, or in the case of *H*. *spinosa*, incomplete lineage sorting due to the strong relationship among their haplotypes. *H*. *spinosa*, has a larger distribution area, where lineages could refuge and re-establish gene flow during unfavorable periods [110–111].

The lineage of *H*. *saxigena* and *H*. *ligustica* is consistently recovered both in plastid and ribosomal trees (Figs 2 and 4), which is interesting as they are species with a stenochoric and partially sympatric distribution. Furthermore, the presence of different common additivities and symplesiomorphies indicate that they could have been differentiated as species recently. Their plastid lineage is related to the *H*. *spinosa* group. We also find the case of *H*. *purpurea* included among haplotypic lineages of *H*. *spinosa*. It is not clear if its origin may be due to horizontal transfer (equal chromosome number) or an incomplete lineage sorting process. This could be favored by the partially sympatric distribution of these species in Sierra Nevada, with *H*. *purpurea* remaining at the top of the mountains, surrounded by *H*. *spinosa*, allowing the existence of gene flow.

Finally, *H*. *pyrenaica*, a narrow endemic, with uncertain affinities seems to be related to *H*. *cochleata* subsp. *cochleata* and *H*. *cochleata* subsp. *baetica* based on the ribosomal evidence, but not at the haplotypic level, where it seems to constitute a separate lineage, and is the nearest haplotype to the sequence of outgroups (Fig 3). This may indicate that it is a case of retention of ancestral haplotypes due to incomplete lineage sorting.

### 4.5 The relevance of alkaline substrates in the tribe Alysseae and *Hormathophylla*

The diversification in the process of speciation resulting from the establishment of the Mediterranean climate (Fig 5) is associated in time and space (in the Baetic ranges) with the colonization of different types of substrate and with the parallel geographical isolation [112–113]. This is clear in the case of species diversification in ITS lineage A (2.17 Ma), all of which are dolomiticolous and restricted to Baetic ranges [25]. Edaphic adaptation has been noted as an important factor in the speciation of different plant groups [95,114–117]. This has already been suggested in other genera belonging to the tribe Alysseae, e.g. *Phyllolepidum* or *Odontarrhena* [11,37]. Adaptation to different substrate types in *Hormathophylla* involved a strong selective pressure that could promote morphological differentiation and the development of reproductive barriers, preventing genetic flow between disjunct divergent populations [118–119]. One of the most remarkable ecological traits associated with the tribe Alysseae is its ability to grow on alkaline, serpentine, dolomite and limestone substrates (S6 Table). This adaptability is present in most of the recently studied lineages [16]. Particularly in the group of genera sister to *Hormathophylla* (1.00 PP; 100 BS) composed of *Lutzia*, *Irania*, *Clastopus*, *Physoptychis*, *Pterygostemon*, *Degenia*, *Acuston*, *Alyssoides*, *Resetnikia*, *Brachypus* and *Fibigia*, many are found on serpentine outcrops [120–121]. This group is sister to another one encompassing *Phyllolepidum* Trinajstic and *Bornmuellera*, which is also prevalent in alkakine substrate (S1 Fig). In total, if we register soil preferences, species growing on alkaline soils constitute a large majority in the tribe, with at least 63 growing on ultramafic, serpentine and volcanic soils and more than 70 on limestone, gypsum and dolomites. Moreover, among all, these genera have species that grow on alkaline substrates with high levels of Mg (both for serpentine and dolomite).

One of the most studied cases of adaptation to extreme edaphic conditions in the Alysseae are nickel hyperaccumulators [11,28–29], which concentrate in two genera (*Odontarrhena* and *Bornmuellera* [122]). Mapping this ecophysiological trait on the ribosomal phylogeny, these species are grouped into two clades along with a high number of taxa able to grow on ultramafic substrates and species tolerant to xeric substrates with high levels of Mg, belonging to 7 out of the 23 genera of Alysseae (i.e., *Bornmuellera*, *Clypeola*, *Fibigia*, *Hormathophylla Meniocus*, *Odontarrhena* and *Physoptychis*). On the other hand, the species exclusively present on dolomite belong to the genera *Hormathophylla*, and *Phyllolepidum*. This relationship between dolomites and serpentines has common factors. The ability to grow on serpentines reveals the adaptation to cope with relatively high concentrations of Ni and other heavy metals. However, in addition to this limiting condition in plant adaptation to ultramafic substrates, there are associated factors developed in the same ecophysiological mechanisms, such as the ability to grow in highly xeric environments [32] in the presence of high levels of Mg [30–31] and a low Ca/Mg ratio, which in many cases are more restrictive than Ni levels [123]. These two characteristics also define soils developed on dolomites [25,124]. The high number of species that grow on particular substrates present in certain lineages seems to adjust to the hypothesis by which some taxa have the availability to thrive on Mg-rich substrates. This seems to be the key preadaptive character in serpentine specialization as demonstrated in other angiosperm families [25,125–126]. The evolutionary relevance between substrate properties and diversification processes have been demonstrated by large-scale studies in the Alps where substrate properties (siliceous or calcareous) are fundamental to explaining modeling of genetic patterns with a large number of species [115].

The adaptation of plant lineages to alkaline or ultramafic substrates has been observed in other Mediterranean regions of the world, in the case of the genera *Lasthenia* Cass. [127] or *Ceanothus* L. [128]. Likewise, this plasticity can be found in other families diversified in the Mediterranean Basin, such as *Cistus* L., Cistaceae [129], or *Onosma* L., Boraginaceae [11], where species associated with a wide range of alkaline substrates show that obligate serpentinophytes share evolutionary ancestors with non-serpentinicolous taxa [11,125], as far as they grow on substrates with high levels of Mg.

There is a strong support in the plastid phylogeny for a derivation of serpentinicolous populations from dolomiticolous in *H*. *longicaulis*, in this case not associated with speciation. However, the transitions between gypsicolous and dolomiticolous habitat is associated with speciation, as in *H*. *cochleata* to *H*. *lapeyrouseana* (Fig 3). Therefore, it seems relevant that the comparative study of plastid genomes through phylogenetic methods could shed light on the evolution of edaphic adaptations. Two species in particular, *H*. *longicaulis* and *H*. *lapeyrouseana*, are especially relevant due to their biedaphic behavior. Such biedaphic behavior has been recorded in other dolomiticolous species such as *Convolvulus compactus* Boiss. or *Jurinea pinnata* DC., [124,130], which are present in serpentine and gypsum, respectively. It seems possible that parallel adaptative mechanisms (in addition to tolerance to environmental stress) work on these three types of substrate [26,131]. As in Cecchi et al. [37], we believe that the Bornmuellera clade, in particular the genus *Hormathophylla*, is an ideal system to perform experimental comparative research on their ability to thrive in metal accumulation conditions and the tolerance to magnesium in plants.

### 4.6 The evolution of trichome morphology in *Hormathophylla*: Taxonomy or ecology?

The observed differences detected among examined taxa, in terms of shape and density of trichomes, raise the question whether these micromorphological fruit and epidermal structures relate to species/lineages or are associated with the ecology and substrate on which the species grows, in all cases xeric habitats (dolomites, serpentines, gypsum, rocky places, etc.). The results of trichome morphological analysis in the Alysseae tribe by Beilstein et al. [10,33] including species of *Hormathophylla* revealed that, considering Brassicaceae, stellate dendritic trichomes exhibit a homoplastic behavior with multiple origins across different lineages in the family. Our results confirm the lack of exclusive trichome types in *Hormathophylla*, and lead to the general conclusion that only closely related species retain similar trichome types.

The presence of the lepidote, stellate and fasciculate trichomes have been linked to functions like atmospheric water absorption, protection from sunlight radiation, and cold insulation in high-mountain habitats [132–133]. In *Hormathophylla*, no strict association was found between the trichome type and substrate beyond the taxonomic boundaries. In contrast within *Hormathophylla*, some types are strongly associated with groups of species, for example, only type I trichomes from lineage A species (*H*. *reverchonii*, *H*. *longicaulis* and *H*. *cadevalliana*, with a basic chromosome number of 7), in which trichome morphology can even be used to distinguish them from the rest of the tribe. The morphological features (i.e., thickness terminal branch, the ratio length/thickness, and the number of primary branching (seven or eight)) can be used as diagnostic characters for these species.

Among the other species belonging to the genus, an indication that trichome morphology agrees with species boundaries is that the type II trichome characterizes the two species *H*. *cochleata* and *H*. *lapeyrouseana*. Both species belong to an allopolyploid lineage derived from a cross of species with a basic chromosome number of x = 7 and x = 8. Interestingly, the trichome size parameters (diameter, thickness of primary rays) of this allopolyploid lineage revealed a significantly more robust structure not found in any other species in the genus, which suggests that this is the result of hybrid vigour traits (S5 Table).

## Supporting information

S1 Table Datasets used in this study with an indication of the content, type of analysis, figures with the resulting topology, and the models of evolution considering AIC and BIC criteria of selection. (DOCX)

S2 Table Voucher information for the 39 taxa belonging to the tribe Alysseae, used for the study of trichomes, studied using scanning electron microscopy (SEM). (DOCX)

S3 Table Comparison of polymorphic sites of the ribosomal sequences obtained from samples of *Hormathophylla*. (XLSX)

S4 Table Comparison of polymorphic sites of the chloroplast regions of the chloroplast regions *trnL-trnF*, *trnT-trnL* and *rpl32-trnL* haplotypes obtained from samples of *Hormathophylla*. (XLSX)

S5 Table Morphological characterization of the different types of trichomes found in *Hormathophylla* genus is shown. (XLSX)

S6 Table List of taxa belonging to the tribe Alysseae and their soil specificity. (DOCX)

S1 Fig Phylogenetic relationships inferred through ML and BI analyses of the 311 samples belonging to the tribe Alysseae, of the ribosomal sequence ITS1-5.8S-ITS2, showed as a majority rule consensus tree. Posterior probability and bootstrap support values are shown beside branches.
(EPS)

S2 Fig Phylogenetic relationships between 246 samples belonging to Alysseae family inferred through ML and BI analysed of the plastid region *ndhF*. Posterior probability values and bootstrap support are shown beside branches.
(EPS)

S3 Fig Phylogenetic relationships between 88 samples belonging to Alysseae family inferred through ML and BI analyses of the concatenated sequence composed of plastid region *ndhF* and ribosomal nuclear ITS1-5.8-ITS2 region. Posterior probability values and bootstrap support are shown beside branches.
(EPS)

S4 Fig Tree generated for estimation of divergence times using BEAST using chloroplastid *ndhF* region. Time scale is shown in millions of years. Error bars show 95% HPD in each node. Values on the right of each node show median in respect with probability in millions of years.
(EPS)

S5 Fig Distribution range of the genera of Alysseae included in the sister group of the genus *Hormathophylla* (0.91 PP/ 61 BS), comprising genera *Lutzia*, *Irania*, *Clastopus*, *Physoptychis*, *Pterygostemon*, *Degenia*, *Acuston*, *Alyssoides*, *Resetnikia*, *Brachypus* and *Fibigia*. Data were obtained from AlyBase (http://www.alysseae.sav.sk/), and the GBIF data-set (data.gbif.org).
(EPS)
