# Supplementary material for: Plant evolution in alkaline magnesium-rich soils: A phylogenetic study of the Mediterranean genus Hormathophylla (Cruciferae: Alysseae) based on nuclear and plastid sequences
Source: PLoS One. 2018 Dec 21;13(12):e0208307. doi: 10.1371/journal.pone.0208307 (PMC6303028; doi:10.1371/journal.pone.0208307)
Supplement: S1 Table — (DOCX) [file pone.0208307.s001.docx]

S1 Table. Datasets used in this study with an indication of the content, type of analysis, figures with the resulting topology, and the models of evolution considering AIC and BIC criteria of selection.

|  |  |  |  |  |  |  |  | **Results** | | |
| --- | --- | --- | --- | --- | --- | --- | --- | --- | --- | --- |
|  |  |  |  |  |  |  |  |  | **Criteria of selection** | |
| **Data Set** | **Sampling** | **Seq. Number** | **Genome** | **Sequenced region** | **Length of alignment** | **Number of polimorphc sites** | **Type of analysis** | **Figure** | **AIC** | **BIC** |
| DS1 | *Hormathophylla +Alysseae*(extended) | 311 | nucleus | ITS | 767 | 522 | BI/ML | S1 | GTR+ Γ+I | GTR+Γ+I |
| DS2 | *Hormathophylla +*Alysseae(extended) | 246 | chloroplast | *ndh*F | 2106 | 1006 | BI/ML | S2 | GTR+ Γ+I | GTR+ Γ+I |
| DS3 | *Hormathophylla*(intraspecific) +outgroups | 96 | choroplast | *trn*Q*-rpl16, trnT-trnL, trnL-trnF,* | 2485 | 131 | BI/ML; TCS | 3,4 | GTR+ Γ+I | GTR+ Γ(+I) |
| DS4 | *Hormathophylla*(intraspecific) +outgroups | 108 | nucleus | ITS | 699 | 147 | BI/ML | 2 | GTR+ Γ+I | GTR+ Γ (+I) |
| DS5 | *Hormathophylla +Alysseae* | 90 | combined | ITS- *ndh*F | 2570 | 909 | BI BEAST | S3 | GTR+ Γ+I | GTR+ Γ+I |
| DS6 | *Hormathophylla +Alysseae*(reduced) | 184 | nucleus | ITS | 696 | 338 | Dating-BEAST | 5 | GTR+ Γ | GTR+ Γ |
| DS7 | *Hormathophylla +Alysseae*(reduced) | 96 | chloropast | *ndh*F | 1999 | 495 | Dating-BEAST | S4 | GTR+ Γ(+I) | GTR+ Γ |
