# Supplementary material for: Plant evolution in alkaline magnesium-rich soils: A phylogenetic study of the Mediterranean genus Hormathophylla (Cruciferae: Alysseae) based on nuclear and plastid sequences
Source: PLoS One. 2018 Dec 21;13(12):e0208307. doi: 10.1371/journal.pone.0208307 (PMC6303028; doi:10.1371/journal.pone.0208307)
Supplement: S2 Table — (DOCX) [file pone.0208307.s002.docx]

S2 Table. Voucher information for the 39 taxa belonging to the tribe Alysseae, used for the study of trichomes, studied using scanning electron microscopy (SEM).

|  |  |
| --- | --- |
| **Species** | **Voucher** |
| *Alyssoides utriculata* | MA-301996 |
| *Alyssum alyssoides* | MA-300508, HUAL-17069 |
| *Alyssum fastigiatum* | HUAL-3536 |
| *Alyssum granatense* | HUAL-13873, HUAL-10564 |
| *Alyssum montanum* | HUAL-15038, HUAL-3482 |
| *Aurinia corymbosa* | MA-803463 |
| *Aurinia leucadea* | MA-692159 |
| *Aurinia sinuata* | MA-49417 |
| *Berteroa incana* | MA-471424 |
| *Berteroa mutabilis* | MA-49683 |
| *Berteroa orbiculata* | MA-417966 |
| *Bornmuellera baldaccii* | MA-555347 |
| *Bornmuellera emarginata* | MA-45307 |
| *Bornmuellera tymphaea* | MA-314083 |
| *Brachypus suffruticosus* | MA-431355 |
| *Clastopus erubescens* | MA-49699 |
| *Clypeola eriocarpa* | MA-4964 |
| *Clypeola jonthlaspi* | MA-49441 |
| *Cuprella antiatlantica* | MA-49591 |
| *Cuprella homalocarpa* | E-00376619 |
| *Fibigia clypeata* | MA-300585 |
| *Hormathophylla cadevalliana* | HUAL-3467, HUAL-1274, MA-228259, HUAL-8248 |
| *Hormathophylla cochleata* subsp. *baetica* | MA-47503, HUAL-3883 |
| *Hormathophylla cochleata* subsp. *cochleata* | MA-303409 |
| *Hormathophylla lapeyrouseana* | MA-303681 |
| *Hormathophylla ligustica* | MA-564248 |
| *Hormathophylla purpurea* | MA-646919 |
| *Hormathophylla reverchonii* | MA-49589 |
| *Hormathophylla saxigena* | MA-49551 |
| *Hormathophylla spinosa* | MA-468032 |
| *Lepidotrichum uechtritzianum* | MA-194697 |
| *Lutzia cretica* | MA-49698 |
| *Meniocus linifolius* | HUAL-17603 |
| *Odontarrhena muralis* | MA-192774 |
| *Odontarrhena serpyllifolia* | HUAL-72, HUAL-14586, HUAL-14600 |
| *Phyllolepidum cyclocarpum* | MA-49382 |
| *Phyllolepidum rupestre* | MA-698734 |
| *Physoptychis gnaphalodes* | MA-430771 |
| *Resetnikia triquetra* | MA-300583 |
