# Supplementary material for: Plant evolution in alkaline magnesium-rich soils: A phylogenetic study of the Mediterranean genus Hormathophylla (Cruciferae: Alysseae) based on nuclear and plastid sequences
Source: PLoS One. 2018 Dec 21;13(12):e0208307. doi: 10.1371/journal.pone.0208307 (PMC6303028; doi:10.1371/journal.pone.0208307)
Supplement: S6 Table — (DOCX) [file pone.0208307.s006.docx]

S6 Table. List of taxa belonging to the tribe Alysseae and their soil specificity.

| **Species** | **type of substrate-soil** |
| --- | --- |
| *Acuston lunaroides* | limestone clifts^40^ |
| *Alyssoides utriculata* | limestone^20^ |
| *Alyssum aizoides* | rocky places^40^ |
| *Alyssum alyssoides* | calcareous^23^, schist^24^and granite^24^ |
| *Alyssum argyrophyllum* | mountain screes^40^ |
| *Alyssum artwinense* | igneous rocky slopes^40^ |
| *Alyssum armenum* | rocky slopes^40^ |
| *Alyssum atlanticum* | high-mountain dry grasslands, rocks and alpine pastures^48^ |
| *Alyssum aurantiacum* | alpine mountain screes and slopes^40^ |
| *Alyssum baumgartnerianum* | limestone screes and slopes^15,40^ |
| *Alyssum bornmuelleri* | screes and steppe^40^ |
| *Alyssum bulbotrichum* |  |
| *Alyssum cacuminum* | alpine screes, rocks and dry grasslands^48^calcareous rocks or schist^24^ |
| *Alyssum caespitosum* | limestone steppe and shale^40^ |
| *Alyssum calycocarpum* | dry, rocky slopes^42^ |
| *Alyssum cephalotum* |  |
| *Alyssum contemptum* | cropland^,^ metamorphic and igneous rocks^40^ |
| *Alyssum corningii* | steppe^40^ |
| *Alyssum cuneifolium* | calcareous^49^ |
| *Alyssum dasycarpum* | serpentine^18^, cultivated ground and steppe^40^ |
| *Alyssum diffusum* | calcareous rocky slopes^52^ |
| *Alyssum doerfleri* | rocks and gravel on limestone^36^ |
| *Alyssum erosulum* | rocky slopes^40^ |
| *Alyssum fastigiatum* | calcareous^24, 48^, dolomite^24, 48^ siliceous^24^ |
| *Alyssum flexicaule* | alpine calcareous screes^48^ |
| *Alyssum foliosum* | calcareous screes^40^ |
| *Alyssum fulvescens* | limestone screes and sand dunes^40^ |
| *Alyssum gallaecicum* | Coastal sand dunes^48^ |
| *Alyssum gmelinii* | calcareous and serpentine rock sand sands^50,55^ |
| *Alyssum granatense* | limestone^24^, schistand granite^24^ |
| *Alyssum gustavssonii* | limestone, rarely schist^42^ |
| *Alyssum harputicum* | steppe^40^ |
| *Alyssum handelii* | mobile screes, rocky and gravelly places on limestone^36^ |
| *Alyssum hirsutum* | disturbed and ruderal habitats, cultivated plant and steppe^40^ |
| *Alyssum idaeum* | mobile and stabilized screes and gravel^,^limestone^36^ |
| *Alyssum lassiticum* | rocky places on limestone^36^ |
| *Alyssum lepidotum* | limestone screes and mountain steppe^40^ |
| *Alyssum lepidoto-stellatum* | calcareous steppe and hills^40^ |
| *Alyssum loiseleurii* | dunes and coastal sands ^24, 48^ |
| *Alyssum lycaonicum* | dry steppe^40,41^ |
| *Alyssum macropodum* | disturbed habitats, cultivated land and chalk slopes calcareous^40^ |
| *Alyssum minutum* | limestone rocky substrates, rarely schist^24^limestone^36^, open places^40^ |
| *Alyssum mouradicum* | rocky slopes open forest^40^ |
| *Alyssum montanum* | calcareous rocks and sands^48^ |
| *Alyssum mouradicum* | limestone^46^ |
| *Alyssum niveum* | calcareous steppe^40^ |
| *Alyssum ochroleucum* | limestone screes and slopes^40^ |
| *Alyssum orophilum* | calcareous rocky slopes^48, 53^ |
| *Alyssum paphlagonicum* | open stony places^40^ |
| *Alyssum pogonocarpum* | serpentine^37^ |
| *Alyssum praecox* | rocky slopes^40^ |
| *Alyssum propinquum* | screes and rocks^40^ |
| *Alyssum pseudomouradicum* | steppe slopes^40^ |
| *Alyssum pulvinare* | rocky places on limestone^36^ |
| *Alyssum repens* | calcareous soils^50, 51^, forests^50^ |
| *Alyssum rhodanense* | granite rocks^48^ |
| *Alyssum siculum* | rocky slopes and pastures limestone^36^ |
| *Alyssum simplex* | limestone^24^, schist and granite^24^, serpentine^15^ |
| *Alyssum smyrnaeum* | fallow fields, slopes, machie^40^ |
| *Alyssum sphacioticum* | mobile limestone screes^36^ |
| *Alyssum spruneri* | serpentine^27^ |
| *Alyssum stapfii* | rocky slopes^40^, serpentine^15^ |
| *Alyssum stribrnyi* | Limestone rocks^47^ |
| *Alyssum strictum* | serpentine^15^, cultivated land and slopes^40^ |
| *Alyssum strigosum* | stony or rocky soils and disturbed hábitats ^40^ |
| *Alyssum sulphureum* | calcareous screes stepps^40^ |
| *Alyssum szovitsianum* | dry open places cultivated land ^40^ |
| *Alyssum taygeteum* | gravelly places, on limestone^36^ |
| *Alyssum tetrastemon* | fallow fields, steppe, screes^40^ |
| *Alyssum thymops* | steppe cultivated ground^40^ |
| *Alyssum trichostachyum* | scrub slopes and forest^40^ |
| *Alyssum trichocarpum* | chalk^40^ |
| *Alyssum turkestanicum* | serpentine ^15,20^, cultivated ground open places^40^ |
| *Alyssum umbellatum Desv.* | cultivated land, rock and screes^40^ |
| *Alyssum wulfenianum* | calcareous rocks^23^ |
| *Alyssum xanthocarpum* | slopes and forest^36,40^ |
| *Aurinia corymbosa* | rocky places on limestone and flysch^36^ |
| *Aurinia gionae* | rocky meadows and ledges, limestone^36^ |
| *Aurinia moreana* | limestone^36^ |
| *Aurinia saxatilis* | limestone^20^, rocky places^40^ |
| *Berteroa incana* | all types of substrate^34^ |
| *Berteroa mutabilis* | banks, walls and rocky slopes ^40^ |
| *Berteroa obliqua* | limestone^36^ |
| *Berteroa orbiculata* | in sands^40^ |
| *Bornmuellera angustifolia* | cited in^40^ |
| *Bornmuellera baldaccii* | serpentine^19,20^ |
| *Bornmuellera cappadocica* | slopes and pastures^40^ |
| *Bornmuellera davisii* | ultramafic^22^ |
| *Bornmuellera dieckii* |  |
| *Bornmuellera emarginata* | serpentine^19^ |
| *Bornmuellera glabrescens* | serpentine |
| *Bornmuellera kiyakii* | serpentine^29,37^ |
| *Bornmuellera tymphaea* | serpentine^19,20^ |
| *Bornmuellera× Leptoplax* |  |
| *Brachypus suffruticosus* | igneous^3,40^ |
| *Clastopus erubescens* | igneous^41^ |
| *Clastopus vestitus* | igneous^41^ |
| *Clypeola aspera* |  |
| *Clypeola cyclodontea* | basic substrates^13^ |
| *Clypeola dichotoma* | volcanic outcrops^41^ |
| *Clypeola eriocarpa* |  |
| *Clypeola jonthlaspi* | indiferent^13^, serpentine ^17^ |
| *Clypeola lappacea* | clay ridges^41^ |
| *Cuprella antiatlantica* | limestone^43^ |
| *Cuprella homalocarpa* | basaltic or siliceous soils^43^ |
| *Degenia velebitica* | limestone^20^ |
| *Fibigia clypeata* | serpentine^3^, calcareous^14^ |
| *Fibigia eriocarpa* | gypsum and calcareous soils, slopes screes^40^ |
| *Fibigia macrocarpa* | rocky slopes^40^ |
| *Galitzkya macrocarpa* | montane alpine rock crevices^39^ |
| *Galitzkya potaninii* | Trans Altai Gobi^39^ |
| *Galitzkya spathulata* | petrophytic steps of mountains and hills in central Asia^39^ |
| *Hormathophylla baetica* | limestone, dolomite^5^ |
| *Hormathophylla cadevalliana* | calcareous,dolomite^5^ |
| *Hormathophylla cochleata* | all type of substrate^33^ |
| *Hormathophylla lapeyrouseana* | gypsum, dolomite (and other types of substrate)^5^ |
| *Hormathophylla ligustica* | limestone ^7^ |
| *Hormathophylla longicaulis* | calcareous, dolomite, serpentine^5^ |
| *Hormathophylla purpurea* | schist, ultramaphic^5^ |
| *Hormathophylla pyrenaica* | limestone^32^ |
| *Hormathophylla reverchonii* | calcareous,dolomite^5^ |
| *Hormathophylla saxigena* | limestone^31^ |
| *Hormathophylla spinosa* | schist, limestone^5^ |
| *Irania umbellata* | limestone^2^ |
| *Lepidotrichum uechtritzianum* | limestone, coastal sands^26,40^ |
| *Lutzia cretica* | limestone^4^ |
| *Meniocus aureus* | cultivated land and steppe^40^ |
| *Meniocus blepharocarpus* | disturbed habitats and steppe^40^ |
| *Meniocus huetii* | disturbed habitats and steppe^40^ |
| *Meniocus linifolius* | calcareous marsh^24^ |
| *Meniocus meniocoides* | serpentine^15^,limestone^40^ |
| *Meniocus stylaris* | negleted soils^40^ |
| *Odontarrhena akamasica* | serpentine^12^ |
| *Odontarrhena alpestris* | serpentine^12^,limestone^20^, calacareous^23^ |
| *Odontarrhena anatolica* | serpentine ^23,30^, calcareous steppes^37^ |
| *Odontarrhena argentea* | limestone^20^andserpentine^23^ |
| *Odontarrhena bertolonii* | serpentine^20,23^ |
| *Odontarrhena borzaeana* | serpentine^20^ |
| *Odontarrhena bracteata* | serpentine^15^ |
| *Odontarrhena callichroa* | serpentine^12, 38^ |
| *Odontarrhena carica* | serpentine^18,23,37,38, 40^ |
| *Odontarrhena cassia* | serpentine^12^ |
| *Odontarrhena chalcidica* | schists and serpentine^16,36^ |
| *Odontarrhena chondrogyna* | serpentine^12^ |
| *Odontarrhena cilicica* | serpentine^12^ |
| *Odontarrhena condensata* | serpentine ^12^,calcareous^23^ |
| *Odontarrhena constellata* | serpentine^12^ |
| *Odontarrhena corsica* | serpentine^23^ |
| *Odontarrhena corymbosoidea* | schist^20^, limestone^20^ |
| *Odontarrhena crenulata* | serpentine^12^ |
| *Odontarrhena cyprica* | serpentine^23^ |
| *Odontarrhena davisiana* | serpentine, igneous^12,23^ |
| *Odontarrhena diffusa* | serpentine^20^ |
| *Odontarrhena discolor* | serpentine, limestone, sandstone^12,37^ |
| *Odontarrhena dubertretii* | serpentine^30^ |
| *Odontarrhena dudleyi* | serpentine^30^ |
| *Odontarrhena eriophylla* | serpentine^12^ |
| *Odontarrhena euboea* | serpentine^20^ |
| *Odontarrhena fallacina* | limestone^20^, serpentine^23,28^ |
| *Odontarrhena filiformis* | steppe open places^40^, serpentine^18^ |
| *Odontarrhena floribunda* | serpentine ^23,30^ |
| *Odontarrhena fragillima* | gravelly places rock crevices limestone^36^ |
| *Odontarrhena gehamensis* | slopes and screes^40^ |
| *Odontarrhena gevgelicensis* | siliceous site^54^ |
| *Odontarrhena giosnana* | serpentine^30^ |
| *Odontarrhena haussknechtii* | alpine on rocky limestone slopes^40^ |
| *Odontarrhena heldreichii* | serpentine^19,20^ , ophiolitic^36^ |
| *Odontarrhena huber-morathii* | serpentine ^23,30^ |
| *Odontarrhena inflata* | facing clay slope^41^ |
| *Odontarrhena kavadarcensis* | siliceous site^54^ |
| *Odontarrhena lesbiaca* | serpentine^37,40^ |
| *Odontarrhena markgrafii* | serpentine^19,25^ |
| *Odontarrhena masmenaea* | serpentine^23,30,37^ |
| *Odontarrhena muralis* | serpentine^12,18,19,20,28,30^_,_ limestone^20^, schist^20^ |
| *Odontarrhena mughlaei* | serpentine^37^ |
| *Odontarrhena nebrodensis* | limestone^20,23,36^ |
| *Odontarrhena obovata* | serpentine^23^, limestone^35^ |
| *Odontarrhena orbelica* | limestone^20^ |
| *Odontarrhena oxycarpa* | serpentine^23,30^ |
| *Odontarrhena pateri* | serpentine^18,30^ |
| *Odontarrhena peltarioidea* | serpentine^18,30^and surrounding areas^23^ |
| *Odontarrhena penjwinensis* | serpentine^12^ |
| *Odontarrhena pinifolia* | serpentine^30^ |
| *Odontarrhena pterocarpa* | serpentine^30,23^ |
| *Odontarrhena robertiana* | limestone^20^, serpentine^20^ |
| *Odontarrhena samarifera* | serpentine^30^ |
| *Odontarrhena serpentina* | serpentine^54^ |
| *Odontarrhena serpyllifolia* | serpentine^12^, calcareous^24^ |
| *Odontarrhena sibirica* | serpentine^20,30,18^ |
| *Odontarrhena singarensis* | serpentine^12^ |
| *Odontarrhena skopjensis* | serpentine^54^ |
| *Odontarrhena smolikana* | serpentine^20^ ophiolitic ^36^ |
| *Odontarrhena syriaca* | serpentine^30^ |
| *Odontarrhena tortuosa* | serpentine^12,18^, calcareous rocks and sands^56^ |
| *Odontarrhena trapeziformis* | serpentine^30^ |
| *Odontarrhena troodi* | serpentine^20^ |
| *Odontarrhena virgata* | serpentine^23,30^ |
| *Phyllolepidium cyclocarpum* | limestone^20^ |
| *Phyllolepidium rupestre* | limestone^20,40^ |
| *Physcoptychis caspica* | rocky slopes^47^ |
| *Physoptychis purpurascens* | serpentine^9,37^and chalk^9^ |
| *Pterygostemon spathulatus* | rocky and stony places^45^ |
| *Resetnikia triquetra* | limestone^1,10^ |

Literature cited:

**1** Prevalek-Kozlina, B., Kostović-Vranjes, V., & D. Slade.1997. *In vitro* propagation of *Fibigia triquetra* (DC.) Boiss., a rare stenoendemic species. *Plant Cell, Tissue and Organ Culture* 51: 141–143.

**2** Isotype of *Farsetia umbellata*. Specimen P00747686. Muséum national d’Histoire naturelle, Paris (France). Collection: Vascular plants (P). Item id: <http://coldb.mnhn.fr/catalognumber/mnhn/p/p00747686> [accessed 22 July 2016].

**3** Çetin,Ö., Duran, A., Martin E., & T.Süleyman. 2011. A taxonomic study of the genus *Fibigia* Medik. (Brassicaceae). *African Journal of Biotechnology* 11(1): 109-119.

**4** Lenton, S. 2010. Cretan Flora. An illustrated guide to the Flora of Creta. *Lutzia cretica*. Website <http://www.cretanflora.com/lutzia_cretica.html> [accessed 22 July 2016].

**5** Küpfer, PH. 1993. *Hormathophylla* Cullen & T.R. Dudley L., *In* S. Castroviejo, et al. [eds.], Flora Iberica. Plantas Vasculares de la Península Ibérica e Islas Baleares*.* vol. 4,. 185-196. C.S.I.C., Madrid, Spain.

**6** Mota, J. F., Medina-Cazorla, J. M., Navarro, F. B., Pérez-García, F. J., Pérez-Latorre, A., Sánchez-Gómez, P., Torres, J. A., Benavente A., Blanca G., Gil, C., Lorite, J., & M. E. Merlo. 2008. Dolomite flora of the Baetic ranges glades (South Spain). *Flora* 203(5): 359-375.

**7** Noble, V. 2008.Typologie des groupementsvégétaux des Alpes d'Azur (Préalpes de Grasse –Alpes-Maritimes) Projet pour unfuturParc Naturel Régional. Conservatoire Botanique National Méditerranéen de Porquerolles. Région PACA, Porquerolles, France.

**8** Morales, C. 2009. *Hormathophylla* Cullen & T.R. Dudley. *In* B. Cabezudo, M. Cueto, C. Fernández López, & C. Morales Torres [eds.], Flora vascular de Andalucía oriental. Vol 3., 103-108. Consejería de Medio Ambiente, Junta de Andalucía, Sevilla, Spain.

**9** Celik, N., Akpulat, H. A., & E. Doenmez. 2007. A new species of *Physoptychis* (Brassicaceae) from central Anatolia, Turkey. *Botanical Journal of the Linnean Society* 154(3): 393-396.

**10** Prevalek-Kozlina, B., Kostović-Vranjes, V., & D. Slade.1997. In vitro propagation of *Fibigia triquetra* (DC.)Boiss., a rare stenoendemic species. *Plant cell, tissue and organ culture* 51(2): 141-143.

**11** Chardot, V., Massoura, S. T., Echevarria, G., Reeves, R. D., &J. L. Morel. 2005. Phytoextraction potential of the nickel hyperaccumulators *Leptoplax emarginata* and *Bornmuellera tymphaea*. *International Journal of Phytoremediation* 7(4): 323-335.

**12** Brooks, R. R., Morrison, R. S., Reeves, R. D., Dudley, T. R., & Y. Akman. 1979. Hyperaccumulation of nickel by *Alyssum* Linnaeus (Cruciferae). *Proceedings of the Royal Society of London B: Biological Sciences* 203(1153): 387-403.

**13** Morales, R. 1993. *Clypeola* L., *In*S. Castroviejo, C. Aedo, M. Laínz, F. Muñoz Garmendia, G. Nieto Feliner, J. Paiva, and C. Benedí[eds.], Flora Iberica. Plantas Vasculares de la Península Ibérica e Islas Baleares*.* vol. 4., 200-203. C.S.I.C., Madrid, Spain.

**14** Fernandes, R.B. 1993. *Fibigia* Medik.*In* S. Castroviejo, C. Aedo, M. Laínz, F. Muñoz Garmendia, G. Nieto Feliner, J. Paiva, and C. Benedí [eds.], Flora Iberica. Plantas Vasculares de la Península Ibérica e Islas Baleares*.* vol. 4., 163-165. C.S.I.C., Madrid, Spain.

**15** Ghaderian, S. M., Mohtadi, A., Rahiminejad, M. R., &A. J. M. Baker. 2007. Nickel and other metal uptake and accumulation by species of *Alyssum* (Brassicaceae) from the ultramafics of Iran.*Environmental Pollution* 145(1): 293-298.

**16** Tsiripidis, I., Papaioannou, A., Sapounidis, V., & E. Bergmeier. 2010. Approaching the serpentine factor at a local scale—a study in an ultramafic area in northern Greece. *Plant and soil* 329(1-2): 35-50.

**17**Hand, R. 2001. Supplementary notes to the flora of Cyprus II. *Willdenowia* 31(2): 383-409.

**18** Altinözlü, H., Karagöz, A., Polat, T., & İ. Ünver. 2012. Nickel hyperaccumulation by natural plants in Turkish serpentine soils. *Turkish Journal of Botany* 36(3): 269-280.

**19** Bani, A., Echevarria, G., Mullaj, A., Reeves, R., Louis Morel, J., & S. Sulçe. 2009. Nickel hyperaccumulation by Brassicaceae in serpentine soils of Albania and northwestern Greece. *Northeastern Naturalist* 16(5): 385-404.

**20** Cecchi, L., Gabbrielli, R., Arnetoli, M., Gonnelli, C., Hasko, A., & F. Selvi. (2010). Evolutionary lineages of nickel hyperaccumulation and systematics in European Alysseae (Brassicaceae): evidence from nrDNA sequence data. *Annals of Botany* 106(5): 751-767.

**21** Naumovski, D. 2005. Germination ecology of seeds of endemic species *Degenia velebitica* (Degen) Hayek (Brassicaceae).*Acta Botanica Croatica* 64(2): 323-330.

**22** Murat, Ü. N. A. L., & L. Behçet. 2007. Flora of Pirreflit Mountain (Van, Turkey). *Turkish Journal of Botany* 31: 193-223.

**23** Mengoni, A., Baker, A. J. M., Bazzicalupo, M., Reeves, R. D., Adigüzel, N., Chianni, E., Galardi, F., Gabbrielli, R., &C. Gonnelli.2003. Evolutionary dynamics of nickel hyperaccumulation in *Alyssum* revealed by ITS nrDNA analysis. *New Phytologist* 159(3): 691-699.

**24** Küpfer, Ph., & G. Nieto Feliner. 1993. *Alyssum* L. *In* S. Castroviejo, C. Aedo, M. Laínz, F. Muñoz Garmendia, G. Nieto Feliner, J. Paiva, and C. Benedí [eds.], Flora Iberica. Plantas Vasculares de la Península Ibérica e Islas Baleares*.* vol. 4., 167-184. C.S.I.C., Madrid, Spain.

**25** Bani, A., Echevarria, G., Mullaj, A., Reeves, R., Louis Morel, J., & S. Sulçe. 2009. Nickel hyperaccumulation by Brassicaceae in serpentine soils of Albania and northwestern Greece.*Northeastern Naturalist* 16(5): 385-404.

**26** Filipova, M. 2016. *Lepidotrichum uechtritizianum* (Bornm.)Vel. United Nations Environment Programme.DEWA/GRID-Geneva. Website http://www.grid.unep.ch/bsein/redbook/txt/lepidotr.htm [accessed 22 July 2016].

**27** Tomović, G. M., Mihailović, N. L., Tumi, A. F., Gajić, B. A., Mišljenović, T. D., &M. S. Niketić. 2013. Trace metals in soils and several Brassicaceae plant species from serpentine sites of Serbia. *Archives of Environmental Protection* 39(4): 29-49.

**28** Jaffré, T., Reeves, R. D., & T. Becquer. 1997. The ecology of ultramafic and metalliferous areas.Proceedings of the 2ndInternationalConference
on SerpentineEcology, Noumea, New Caledonia, July.

**29** Reeves, R. D., Adıgüzel, N., & A. J. Baker. 2009. Nickel hyperaccumulation in BornmuellerakiyakiiAytaç&Aksoy and associated plants of the Brassicaceae from Kızıldağ (Derebucak, Konya-Turkey). *Turkish Journal of Botany* 33(1): 33-40.

**30** Reeves, R. D., & and N. Adigüzel. 2008. The nickel hyperaccumulating plants of the serpentines of Turkey and adjacent areas: a review with new data. *Turkish Journal of Biology* 32(3): 143-153.

**31** Küpfer, PH. 1974.Recherchessurlesliensdeparentéentre lafloreorophiledesAlpesetcelle *des* Pyrénées*.* Boissiera 23: 211

**32** Küpfer, PH. 1974. Recherchessurlesliensdeparentéentre lafloreorophiledesAlpesetcelle *des* Pyrénées.Boissiera 23: 207

**33** Küpfer, PH. 1974. Recherchessurlesliensdeparentéentre lafloreorophiledesAlpesetcelle *des* Pyrénées. Boissiera 23: 1-322

**34** Jacobs, J., & J. Mangold. 2008. Plant fact sheetfor hoary Alyssum (*Berteroaincana* (L.)DC.). USDA-Natural Resources Conservation ServiceWebsite <https://plants.usda.gov/factsheet/pdf/fs_bein2.pdf> [accessed 22 July 2016].

**35** Nawrocki, T., Fulkerson, J., & M. Carlson. 1997. *Alyssum obovatum* (C.A. Mey.) Turcz. Alaska Rare plant field Guide. Alaska Center for Conservation Science.University of Alaska Anchorage.Website<http://aknhp.uaa.alaska.edu/big-files/botany/Alaska_Rare_Plant_Field_Guide/Alyssum_obovatum.pdf> [accessed 22 July 2016].

**36** Strid, A. [ed.], 1986. Mountain flora of Greece.vol. 1, Cambridge University Press, Cambridge, UK.

**37** Kurt, L., Ozbey, B. G., Kurt, F., Ozdeniz, E., & A. Bolukbasi. 2013. Serpentine Flora of Turkey. *Biological Diversity and Conservation* 6: 134-152.

**38** Prasad, M. N. V. 2005. Nickelophilous plants and their significance in phytotechnologies. *Brazilian Journal of Plant Physiology* 17(1): 113-128.

**39** Wesche, K., Jäger, E. J., von Wehrden, H., & R. Undrakh. 2005. Status and distribution of four endemic vascular plants in the Gobi Altai. *Mongolian Journal of Biological Sciences*3: 3-11.

**40** Dudley, T. R. 1965. *Alyssum* L. .*In* P. H. Davis [ed.],Flora of Turkey and the East Aegean Islands. vol. 1., 362–409. Edinburgh University Press.Edinburgh, UK.

**41** Herbarium catalogue of the Royal Botanic Garden of Edinburgh.

*Clastopuserubescens* <http://data.rbge.org.uk/herb/E00328165>

*Clastopusvestitus* <http://data.rbge.org.uk/herb/E00328297>

*Alyssum lycaonicum* <http://data.rbge.org.uk/herb/E00328178>

*Clypeoladichotoma* <http://data.rbge.org.uk/herb/E00376764>

*Clypeolalappacea* <http://data.rbge.org.uk/herb/E00376778>

*Odontarrhena inflata* <http://data.rbge.org.uk/herb/E00379939>

[accessed 22 July 2016].

**42** Tutin, T. G.1980.*Floraeuropaea*.Cambridge University Press, Cambridge, UK.

**43** Španiel, S., Kempa, M., Salmerón-Sánchez, E., Fuertes-Aguilar, J., Francisco Mota, J., Al-Shehbaz, I.A., German, D.A., Olšavská, K., Šingliarová, B., Zozomová-Lihová, J. & K. Marhold. 2015. AlyBase – database of names, chromosome numbers, and ploidy levels of Alysseae (Brassicaceae), with a new generic concept of the tribe. *Plant Systematics and Evolution* 301: 2463–2491.

**44** Roussakova, V. 2015. *Alpine and sub-alpine closed calcareous grasslands.In* V. Velchev [ed.], Red data book of Bulgaria. Extinct, threatened, and rare plants and animals.Plants, vol. 1, 448.Digital edition.Website <http://e-ecodb.bas.bg/rdb/en/vol3/23e4.html>[accessed 22 July 2016].

**45** Komarov, V. L. [ed],1970. Flora of the USSRvol.8.Smithsonian Institution Libraries, Washington, D.C., USA.

**46** Efe, R., Cravins, G., & M. Ozturk [eds.], 2009.Natural Environment and Culture in the Mediterranean Region.Cambridge Scholars Publishing, Cambridge, UK.

**47** Slaby P. 2016. Rock Garden plants Database. Website <http://flora.kadel.cz/g/kvCard.asp-Id=18091.htm> [accessed 22 July 2016].

**48** Zozomová-Lihová, J., Marhold, K., & S. Španiel. 2014. Taxonomy and evolutionary history of *Alyssum montanum* (Brassicaceae) and related taxa in southwestern Europe and Morocco: Diversification driven by polyploidy, geographic and ecological isolation. *Taxon*, *63*(3), 562-591.

**49** Pignatti, S. 1982. Flora d'Italia. Vol. 1. Edagricole,Bologna, Italy. 732 p.

**50** Španiel, S., Marhold, K., Filová, B., & J. Zozomová-Lihová. 2011. Genetic and morphological variation in the diploid–polyploid *Alyssum montanum* in Central Europe: taxonomic and evolutionary considerations. *Plant Systematics and Evolution* 294(1-2), 1.

**51** Alexandru Badarau, A. 2017. Nature of Transilvania. Website <http://www.floraofromania.transsilvanica.net/flora%20of%20romania/ac%20IX%20801-900%20dan%20turtureanu/Copy%20(13)%20of%20Copy%20of%20species.htm> [accessed 22 June 2017].

**52** Španiel, S., Zozomová-Lihová, J., Passalacqua, N.G., & K. Marhold.2012. Infraspecific classification of *Alyssum diffusum* (Brassicaceae) in Italy. *Willdenowia* 42: 37-56.

**53** Španiel, S., Marhold, K., Passalacqua, N.G., & J. Zozomová-Lihová. 2011. Intricate variation patterns in the diploid-polyploid complex of*Alyssum montanum-A. repens* (Brassicaceae) in the Apennine peninsula: evidence for long-term persistence and diversification. *American Journal of Botany* 98: 1887-1904.

**54** Micevski, K. 1994. Sect. *Odontarrhena* (C.A.Meyer) Koch kaj rodot *Alyssum* L. (Cruciferae) vo florata na Republika Makedonija. *Prilozi, Oddelenie za Bioloshki i Meditsinski Nauki. Makedonska Akademija na Naukite i Umetnostite.* 15: 41-58.

**55** Španiel, S., Marhold, K., Thiv, M., & J. Zozomová-Lihová. 2012. A new circumscription of *Alyssum montanum* ssp. *montanum*and *A. montanum* ssp. *gmelinii* (Brassicaceae) in Central Europe:Molecular and morphological evidence. *Botanical Journal of the Linnean Society* 169: 378-402.

**56** Goliašová, K. 2002. *Alyssum* L. In: Goliašová, K., Šípošová, H. (eds), Flóra Slovenska 5/4. Veda, Bratislava, pp 469–493.
